# The Antiviral Activity of the Lectin Griffithsin against SARS-CoV-2 Is Enhanced by the Presence of Structural Proteins

**DOI:** 10.3390/v15122452

**Published:** 2023-12-18

**Authors:** Arjan Bains, Kathryn Fischer, Wenyan Guan, Patricia J. LiWang

**Affiliations:** 1 Chemistry and Biochemistry, University of California Merced, 5200 North Lake Rd., Merced, CA 95343, USA; abains5@ucmerced.edu; 2 Quantitative and Systems Biology, University of California Merced, 5200 North Lake Rd., Merced, CA 95343, USA; kfischer4@ucmerced.edu; 3 Materials and Biomaterials Science and Engineering, University of California Merced, 5200 North Lake Rd., Merced, CA 95343, USA; wguan3@ucmerced.edu; 4 Molecular Cell Biology, Health Sciences Research Institute, University of California Merced, 5200 North Lake Rd., Merced, CA 95343, USA

**Keywords:** COVID-19, SARS-CoV-2, DC-SIGN, Griffithsin, M protein, trans-infection, lectin, C-type lectin receptor, prophylaxis, structural proteins

## Abstract

Although COVID-19 transmission has been reduced by the advent of vaccinations and a variety of rapid monitoring techniques, the SARS-CoV-2 virus itself has shown a remarkable ability to mutate and persist. With this long track record of immune escape, researchers are still exploring prophylactic treatments to curtail future SARS-CoV-2 variants. Specifically, much focus has been placed on the antiviral lectin Griffithsin in preventing spike protein-mediated infection via the hACE2 receptor (direct infection). However, an oft-overlooked aspect of SARS-CoV-2 infection is viral capture by attachment receptors such as DC-SIGN, which is thought to facilitate the initial stages of COVID-19 infection in the lung tissue (called trans-infection). In addition, while immune escape is dictated by mutations in the spike protein, coronaviral virions also incorporate M, N, and E structural proteins within the particle. In this paper, we explored how several structural facets of both the SARS-CoV-2 virion and the antiviral lectin Griffithsin can affect and attenuate the infectivity of SARS-CoV-2 pseudovirus. We found that Griffithsin was a better inhibitor of hACE2-mediated direct infection when the coronaviral M protein is present compared to when it is absent (possibly providing an explanation regarding why Griffithsin shows better inhibition against authentic SARS-CoV-2 as opposed to pseudotyped viruses, which generally do not contain M) and that Griffithsin was not an effective inhibitor of DC-SIGN-mediated trans-infection. Furthermore, we found that DC-SIGN appeared to mediate trans-infection exclusively via binding to the SARS-CoV-2 spike protein, with no significant effect observed when other viral proteins (M, N, and/or E) were present. These results provide etiological data that may help to direct the development of novel antiviral treatments, either by leveraging Griffithsin binding to the M protein as a novel strategy to prevent SARS-CoV-2 infection or by narrowing efforts to inhibit trans-infection to focus on DC-SIGN binding to SARS-CoV-2 spike protein.

## 1. Introduction

The COVID-19 pandemic remains a significant challenge for society, with over 760 million cases and about 6.95 million deaths globally as of August 2023 [1]. While the development of several COVID-19 vaccines has alleviated the severity of the pandemic, COVID-19 cases persist, with about 10,000 COVID-19 hospitalizations occurring weekly nationwide in the United States throughout the months of May to July of 2023 [1,2,3,4]. In addition to the ease of transmission of the coronaviral virion, the SARS-CoV-2 spike protein undergoes immune escape quite readily, with studies demonstrating that the neutralizing activities of plasma samples taken from individuals who received either the Pfizer or Moderna vaccinations were significantly less effective against several mutant strains of SARS-CoV-2, including the UK (B1.1.7/501Y.V1), South Africa (B.1.351/501Y.V2), and Brazil (P.1) strains [2,3,5]. More concerningly, the most recent Omicron strains display numerous mutations in their spike protein sequences, which have allowed them to establish breakthrough infections in patients who have received multiple booster doses of COVID-19 vaccines [6,7]. These breakthroughs have prompted the development of multiple booster vaccines against the Omicron strain virus, although the mutation rate of SARS-CoV-2 has necessitated the development and subsequent phasing out of boosters against Omicron BA.1, Omicron BA.4/5, and Omicron XBB.1.5 [8,9,10,11]. Recently, the newest COVID-19 strain, BA.2.86/Pirola, was detected in July of 2023, and it has since expanded to at least seven countries [12]. Its numerous spike mutations and widespread transmission (despite its modest immune evasion capabilities) serve as a reminder that it is still necessary to develop new boosters in preparation for future COVID-19 variants [12,13]. Coupled with breakthrough infections, vaccination is also not always 100% protective or suitable for all patients: some immunodeficient individuals appear to show a drastically reduced capability to produce antibodies against Omicron strains, even with multiple vaccinations [14,15]. Complicating this, there is still robust global vaccine hesitancy, with reluctance growing amongst new parents and younger age groups in several countries around the world [16]. Taken together, all of this indicates it is still valuable to explore preventative treatments to curtail the spread of SARS-CoV-2. Indeed, vaccine companies like AstraZeneca and Merck are currently developing prophylactic treatments for SARS-CoV-2, such as Evusheld and LAGEVRIO/molnupiravir, respectively [17,18].

Griffithsin is a small (~12.8 kilodalton), dimeric, red algae-derived lectin that displays inhibitory activity against a wide variety of viruses, ranging from Human Immunodeficiency Virus (Retroviridae family) to Herpes Simplex Virus (Herpesviridae family), Japanese Encephalitis Virus (Flaviviridae family), Human Papillomavirus (Papillomaviridae family), and other Coronaviridae family viruses including MERS-CoV and SARS-CoV [19,20,21]. Additionally, several studies have been published exploring Griffithsin’s efficacy against SARS-CoV-2, with half-maximal inhibitory concentrations ranging from approximately 5 nM to 3000 nM (Table S1). Each Griffithsin monomer contains three saccharide-binding sites with a predominant affinity for mannose glycans [22,23]. Viral glycoproteins typically present ample mannose sugars that can act as binding sites for Griffithsin, thereby impeding viral entry and infectivity [19]. Griffithsin can be synthesized economically either in plants or bacteria, has been shown to have low toxicity in mammals, can withstand temperatures up to 78 °C, maintains inhibitory capability after several weeks of exposure to 50 °C, can withstand at least five freeze–thaw cycles, and survives exposure to a variety of acid and base solutions for at least 24 h [24,25,26,27]. Moreover, a recent clinical trial showed that Griffithsin is not absorbed into systemic circulation when it is formulated into a gel, indicating that, at the very least, Griffithsin is safe for topical application in humans and, thus, an expedient protein to adapt for use as an anti-COVID-19 prophylactic [25,26,27,28].

A complication is that Wild-Type Griffithsin (WT-Grft) has been shown to undergo spontaneous oxidation at the Methionine 78 residue (M78) when exposed to biological environments [24]. To circumvent this, recent work has been carried out to show that mutating the moiety M78 to Glutamine yields a variant of Griffithsin—called M78Q-Griffithsin (Q-Grft)—which exhibits many of the same chemical and inhibitory properties as Wild-Type Griffithsin while resolving the complications that could arise from oxidation [24,29]. Importantly, Q-Grft is also in clinical trials as a prophylactic nasal spray against SARS-CoV-2 [30].

Another facet of Griffithsin’s use as a prophylactic agent is the theorized importance of Griffithsin’s ability to cross-link target proteins together. As a bivalent sugar-binding protein, evidence from several studies strongly suggests that Griffithsin is capable of binding to epitopes from two separate HIV-1 gp120 proteins at the same time, causing aggregation of the gp120 proteins on the surface of the viral particle and possibly also the aggregation of HIV-1 viruses [26,31]. Removing the sugar-binding sites from one of the two dimeric subunits to prevent bivalent binding significantly impairs WT-Grft’s ability to inhibit HIV pseudovirus despite only mildly diminishing the affinity of Griffithsin for HIV-1 gp120 [31,32]. Other studies show Wild-Type Griffithsin causes aggregation of flagellated unicellular pathogens [33]. Dimeric Griffithsin and other dimeric lectins, such as Cyanovirin-N, are also shown to cause yeast cells to agglutinate, thereby lending further credence to the idea that the inhibitory capabilities of these lectins are tied to their ability to cross-link target proteins [33,34].

To our knowledge, with the exception of one recent paper, the current existing literature exploring Griffithsin’s in vitro inhibitory capabilities against SARS-CoV-2 has solely utilized WT-Grft [35,36,37,38,39]. Although one study observed an approximately 60% stronger SARS-CoV-2 pseudoviral inhibition for M78Q-Grft when compared to WT-Grft, the two constructs used in the publication differed slightly, with the WT-Grft construct retaining a Hexa-histidine tag and an enterokinase cut site [40]. In this study, we first aimed to assess whether the M78Q mutation altered Griffithsin’s ability to act as an anti-SARS-CoV-2 entry inhibitor. We then endeavored to ascertain whether Griffithsin needed to have a bivalent binding capability in order to retain potency against SARS-CoV-2 hACE2-mediated entry.

In the several studies that have come out assessing WT-Grft’s potential use as an anti-SARS-CoV-2 prophylactic, Griffithsin tends to show a half-maximal inhibitory concentration against nCov-19 spike pseudotyped lentivirus in the ~300 nM to ~2000 nM range, which is about an order of magnitude less potent than Griffithsin’s inhibitory capabilities against other viruses like SIV, Hepatitis C, and Japanese Encephalitis Virus, and several orders of magnitude less potent against HIV [35,36]. In some of the same studies that assessed WT-Grft’s antiviral activity against SARS-CoV-2 pseudotyped lentivirus, it was also tested on replication-competent SARS-CoV-2 coronaviral particles [37,38,39]. Interestingly, WT-Grft seemed to consistently exhibit more efficient inhibition of authentic SARS-CoV-2 coronavirus, with a half-maximal inhibitory concentration ranging from 33.2 nM to 100.1 nM (Table S1) [37,38,39]. To our knowledge, no group has explored potential reasons why Griffithsin appears to exhibit higher antiviral potency against genuine replication-competent SARS-CoV-2 virus when compared to SARS-CoV-2 pseudotyped lentivirus.

There are several possible explanations for why there is a disparity in WT-Grft inhibition between lentiviral pseudovirus and genuine SARS-CoV-2 coronaviral particles, including differences in assay read-out (e.g., luciferase signal versus qRT-PCR or viral cytopathic effect), using single-round virus versus replication-competent virus, variations in viral MOI, or differences in viral architecture.

In this work, we focused on whether differences in viral architecture between lentiviral pseudovirions and coronavirus particles could explain the variations in Griffithsin’s ability to prevent viral infection.

HIV and other lentiviruses express approximately 7–14 glycoproteins on the surface of a 100 nm diameter membrane-encapsulated virus [41,42]. Other than these glycoproteins (which dictate viral tropism), the surfaces of lentiviruses are essentially bare of other virus-specific structural features. SARS-CoV-2 coronaviral virions share many features with lentivirus, also being ~100 nm membrane-encapsulated viruses that express 11–41 spike proteins (S) that dictate viral tropism and mediate the infection process [43,44,45]. But the novel coronavirus nCoV-19 genome expresses several other structural proteins that are packaged with the SARS-CoV-2 virion. These include the nucleocapsid protein (N), the envelope protein (E), and the membrane protein (M). As in other coronaviruses, all three structural proteins interact with each other and the spike to facilitate viral budding, protein transport through the Endoplasmic Reticulum, and viral attachment [46,47,48,49,50]. More importantly, the E and M proteins are glycosylated surface proteins, so they could plausibly be recognized by Griffithsin and contribute to the inhibition of viral infection.

We wished to ascertain whether the additional SARS-CoV-2 structural proteins M, N, and E could be responsible for the apparent increased susceptibility of the coronaviral virions to Wild-Type Griffithsin-based inhibition.

Finally, although the Human Angiotensin-converting Enzyme 2 (hACE2) has been solidified as the required receptor to facilitate SARS-CoV-2 viral entry, the SARS-CoV-2 virion is known to be able to infect cells that only modestly express hACE2 [51,52]. This is perhaps best exemplified by how, although COVID-19 presents as a respiratory illness, the human respiratory system has low hACE2 expression [48,51,53,54,55]. While this initially perplexed researchers, it has now been widely accepted that cell surface attachment receptors are capable of binding to the SARS-CoV-2 virion and facilitating efficient viral infection of cells that have poor hACE2 expression [54,56,57,58]. Among these attachment receptors, lectin receptors such as Dendritic Cell-Specific Intercellular Adhesion Molecule-3-Grabbing Non-integrin (DC-SIGN) have been known to enhance the infectivity of a variety of viruses, including human T-cell lymphotropic virus type 1, Enterovirus 71, Dengue Virus, and HIV-1 [33,34,59,60,61,62]. The consensus is that DC-SIGN receptor-mediated trans-infection is crucial in facilitating the initial infection of respiratory tissue in the context of COVID-19 [54,56].

Since DC-SIGN preferentially binds to the same high-mannose glycans that Griffithsin binds to, we hypothesized that Griffithsin could act as a competitive inhibitor against DC-SIGN-mediated trans-infection [19,20,25,28,63].

Overall, in this publication, we examined how the structural features of Griffithsin affected its ability to prevent SARS-CoV-2 pseudotyped lentiviral infection. Specifically, we compared how the M78Q mutation and the cross-linking activity of Griffithsin affect SARS-CoV-2 spike-mediated pseudovirus infection. We also compared how virions that expressed either the additional coronaviral structural proteins affected both virus susceptibility to Griffithsin inhibition and DC-SIGN-mediated trans-infection. It is our hope that this work will assist with the development of Griffithsin variants and prophylactic protocols that can be rapidly deployed against future SARS-CoV-2 strains.

## 2. Materials and Methods

### 2.1. DNA Construction

The gene encoding the Wild-Type Griffithsin (WT-Grft) protein (with an Alanine at position 31 to substitute a non-standard amino acid and an N-terminal 6xHis tag) was cloned into the pET15-b expression vector (Novagen, Madison, WI, USA) between the NcoI and BamHI restriction sites as described previously [22,23].

The gene sequence for M78Q Griffithsin (Q-Grft) with an N-terminal 6xHis tag and a Methionine to Glutamine substitution at position 78 was ordered from Twist Biosciences already inserted into the pET28b vector between the NcoI and XhoI restriction sites.

The gene sequence for Griffithsin–linker–Griffithsin One-armed (GLG-3A) was created by first ordering the gene sequence for M78Q Griffithsin with an N-terminal 6xHis tag and a C-terminal linker with a BamHI site inserted into the pET28b vector between the NcoI and XhoI restriction sites (Twist Bioscience, South San Francisco, CA, USA). Then, the gene sequence for M78Q Griffithsin with an N-terminal BamHI site and Aspartate to Alanine mutations at positions 30, 70, and 112 was inserted into the pET28b vector between the NcoI and XhoI restriction sites (again from Twist Bioscience). Both vectors were then digested with BamHI and XhoI. The M78Q Griffithsin sequence with D30A/D70A/D112A mutations was then inserted into the vector with the N-terminal 6xHis tagged M78Q Griffithsin sequence to yield a pET28b vector with N-terminal 6xHis tagged M78Q Griffithsin–linker–M78Q Griffithsin D30A/D70A/D112A. The linker sequence is as follows: SSSGGGGSGGGSSSGS.

The gene encoding for Monomeric Griffithsin protein (with a 1GS insertion at positions 18 and 19, and the Leucine at position 2 substituted for a Serine) with an N-terminal 6xHis tag was inserted into the pET28b vector between the NcoI and XhoI restriction sites (again from Twist Bioscience).

### 2.2. Protein Production and Purification

All proteins were produced as described previously [22]. Briefly, plasmids of Griffithsin variants described above were transformed into BL21-Gold (DE3) competent cells (Agilent Technologies Catalog # 200131, Santa Clara, CA, USA). Transformed cells were used to inoculate 2-L culture flasks containing 1 L of M9 minimal medium. Once flask medium reached an optical density at 600 nm (OD600) of 0.50 to 0.75, protein production was induced with 0.6 mM of isopropyl β-d-1-thiogalactopyranoside (IPTG, Millipore Sigma OmniPur–Calbiochem, Catalog # 5820, Burlington, MA, USA) and allowed to further incubate at 37 °C for 6 to 10 h.

After incubation, cells were harvested at 6000× *g* for 10 min, and the pellet was resuspended in Resuspension Buffer (6 M Guanidine hydrochloride, 200 mM NaCl, 10 mM Benzamidine, and 50 mM Tris, pH 8). The solution was French-pressed two to three times at 16,000 lb/in^2^ and then centrifuged at 13,500× *g* for 1 h. The soluble portion was loaded onto a Nickel chelating column (Qiagen Catalog # 30210, Germantown, MD, USA) equilibrated with the same Resuspension Buffer. The Nickel columns were washed with Washing Buffer (6 M Guanidine hydrochloride, 200 mM NaCl, and 50 mM sodium phosphate, pH 7) to remove nonspecifically bound proteins. Bound Griffithsin was eluted from columns with Elution Buffer (6 M Guanidine hydrochloride, 200 mM NaCl, and 50 mM sodium acetate, pH 3). Griffithsin proteins were refolded by dropwise addition into 10× greater volume of chilled refold buffer (550 mM L-Arginine, 200 mM NaCl, 50 mM Tris, 1 mM EDTA, pH 8). The protein was allowed to refold for 12 h at 4 °C before the solution was dialyzed twice in 4 Liters of 150 mM NaCl, 20 mM Tris, and pH 8 at 4 °C for 6–12 h each. The protein solution was then dialyzed four times against 4 Liters of 50 mM NaCl, 10 mM Tris, and pH 8 at 4 °C for 6–12 h each.

Proteins were then purified on a C4 reversed-phase chromatography column (Vydac, Hesperia, CA, USA). The Griffithsin constructs were purified on a gradient of water with 0.1% Trifluoroacetic Acid as the counterion (Buffer A) to Acetonitrile with 0.1% Trifluoroacetic Acid as the counterion (Buffer B). The loading solution for the C4 column was 92% Buffer A, 8% Buffer B. Griffithsin constructs tended to elute at approximately 70% Buffer A, 30% Buffer B. After C4 reversed-phase chromatography, elution fractions containing Griffithsin were pooled and lyophilized in a Labconco freeze-dry system for long-term storage. All samples were analyzed by sodium dodecyl sulfate-polyacrylamide gel electrophoresis (SDS-PAGE) at each step of purification to confirm the proper size of the specific Griffithsin construct.

### 2.3. Nuclear Magnetic Resonance (NMR) Spectroscopy

To isotopically label Griffithsin variants, proteins were expressed in M9 minimal media with ^15^NH_4_Cl as the sole nitrogen source following the protocol described above. After proteins were lyophilized, the Wild-Type Griffithsin, M78Q Griffithsin, and Griffithsin–linker–Griffithsin One-armed were resuspended in 20 mM sodium phosphate and pH 7 buffer. Monomeric Griffithsin did not efficiently dissolve in pH 7 buffer, so it was initially resuspended in 20 mM sodium phosphate, pH 2.5 buffer. This was then diluted with an equal volume of 20 mM sodium phosphate and pH 9 buffer to obtain a final pH that varied between 6.62 and 6.96.

The sample of protein was then taken to 5% D_2_O and 2,2-dimethyl-2-silapentane-5-sulfonic acid (DSS, Cambridge Isotope Laboratories Inc., Catalog # DLM-32-10, Tewksbury, MA, USA) was added for calibration. Spectra were collected at 25 °C on a four-channel 600-MHz Bruker Avance III spectrometer. Data were processed using NMRPipe, as described previously [31].

### 2.4. Cell Lines

All cell lines were maintained in T-75 flasks (Stellar Scientific Cat # SKU:TC30-120, Baltimore, MD, USA) placed in a 37 °C humidified incubator (ThermoFisher NAPCO Series 8000DH CO_2_ Incubator, Catalog # 7003584, Waltham, MA, USA) with 4.5% CO_2_. These cells include following:

- HEK-293FT (Homo sapiens, embryonic kidney cells—a generous gift from Dr. David Gravano, University of California, Merced);
- HEK-293T cells expressing Human Angiotensin-converting Enzyme 2/hACE2 (Homo sapiens, embryonic kidney cells—obtained through BEI Resources, NIAID, NIH: Human Embryonic Kidney cells (HEK-293T) expressing Human Angiotensin-converting Enzyme 2, HEK-293T-hACE2 cell line, NR-52511);
- 3t3 Wild-Type cells (Mus musculus, mouse embryonic fibroblasts—obtained through the NIH HIV Reagent Program, Division of AIDS, NIAID, NIH: NIH-3T3 cells, ARP-9946; contributed by Drs. Thomas D. Martin and Vineet N. KewalRamani);
- 3t3 DC-SIGN+ cells (Mus musculus, mouse embryonic fibroblasts—obtained through the NIH HIV Reagent Program, Division of AIDS, NIAID, NIH: NIH 3T3 DC-SIGN+ cells, ARP-9947; contributed by Drs. Thomas D. Martin and Vineet N. KewalRamani);
- Raji Wild-Type cells (Homo sapiens, Epstein–Barr Virus (EBV)-positive Burkitt lymphoma line originally obtained from the American Type Culture Collection (ATCC); obtained through the NIH HIV Reagent Program, Division of AIDS, NIAID, NIH: Raji cells, ARP-9944; contributed by Drs. Li Wu and Vineet N. KewalRamani);
- Raji DC-SIGN+ cells (Homo sapiens, Epstein–Barr Virus (EBV)-positive Burkitt lymphoma line originally obtained from the American Type Culture Collection (ATCC); obtained through the NIH HIV Reagent Program, Division of AIDS, NIAID, NIH: Raji cells, ARP-9945; contributed by Drs. Li Wu and Vineet N. KewalRamani).

HEK-293FT cells and HEK-293T hACE2+ cells were cultured as adherent monolayers in T-75 flasks maintained in 293 medium. The 293 medium is Dulbecco’s Modified Eagle Medium (DMEM) (ThermoFisher Catalog # 11965-092, Waltham, MA, USA) supplemented with 25 mM HEPES (ThermoFisher Catalog # 11344041, Waltham, MA, USA), 2 mM L-glutamine (R&D Systems Catalog # R90010, Minneapolis, MN, USA), 250 μg mL^−1^ G418 Sulfate (Corning Life Sciences Catalog # 30-234-CI, Tewksbury, MA, USA), and 10% Fetal Bovine Serum (R&D Systems Catalog # S11150, Minneapolis, MN, USA).

3t3 cells were selected as a capture cell line because they are not permissive to SARS-CoV-2 pseudoviral infection, nor do they highly express lectin receptors [64,65]. 3t3 Wild-Type cells and 3t3 DC-SIGN+ cells were cultured as adherent monolayers in T-75 flasks maintained in 3t3 medium. 3t3 medium is DMEM supplemented with 2 mM GlutaMAX (ThermoFisher Cat # 35050-061, Waltham, MA, USA), 100 U mL^−1^ of Penicillin–Streptomycin solution (Cytiva HyClone Cat # SV30010, Marlborough, MA, USA), and 10% Fetal Bovine Serum (R&D Systems Cat # S11150, Minneapolis, MN, USA).

Raji cells were selected because the paired Raji Wild-Type and Raji DC-SIGN+ cell lines have been a workhorse strain in viral trans-infection assays [66,67,68,69]. Both Raji Wild-Type cells and Raji DC-SIGN+ cells were maintained in upright T-75 flasks as a suspension in Raji medium. Raji medium is Roswell Park Memorial Institute 1640 Medium (RPMI) (ThermoFisher Cat # 11875-085, Waltham, MA, USA) supplemented with 100 U mL^−1^ of Penicillin–Streptomycin solution (Cytiva HyClone Cat # SV30010, Marlborough, MA, USA) and 10% Fetal Bovine Serum (R&D Systems Cat # S11150, Minneapolis, MN, USA).

When the adherent cell lines (HEK-293FT, HEK-293T hACE2, 3t3 Wild-Type, and 3t3 DC-SIGN+ cells) reached 80–95% confluency, they were detached with 0.05% Trypsin-EDTA (ThermoFisher Cat # 25300-062, Waltham, MA, USA) and were passaged 1:10 with fresh medium suitable for each cell type (i.e., HEK-293 cells received 293 medium and 3t3 cells received 3t3 medium). Trypsin was not permitted to exceed a contact time of 5 min for any cell line.

When Raji Wild-Type cells or Raji DC-SIGN+ cells reached a density of 5 × 10^6^ cells per mL, cells were split by diluting 10-fold in fresh Raji medium.

All cell lines were used within 1.5 months of being revived to minimize the likelihood that cell protein expression changed.

### 2.5. Pseudovirus Production

SARS-CoV-2 Wuhan nCov-19 strain (NCBI Reference Sequence: NC_045512.2) spike pseudotyped lentiviral virions were produced as previously described [70]. Briefly, HEK-293FT cells were maintained in 293 media and seeded at a density of approximately 2.5 × 10^6^ cells in 8–10 mL of 293 media into a 100 mm tissue culture Petri dish or approximately 0.2 × 10^6^ cells in 2–3 mL of 293 media into a 6-well tissue culture plate (batches of virus were made in either of the two plate types). Cells were allowed to recover for 12–16 h in a humidified incubator at 37 °C and 5% CO_2_.

The next day, Petri dishes or 6-well plates were retrieved, and media were replaced with either 8 mL or 3 mL of fresh 293 medium, respectively. The Petri dishes or 6-well plates were returned to the humidified incubator, and lentiviral pseudotyping vectors were prepared as described by Crawford et al., 2020 [71]. Briefly, for 100 mm tissue culture Petri dishes, the following were mixed with 900 µL of serum-free DMEM in a 1.5 mL microcentrifuge tube: 5 μg of lentiviral backbone Luciferase-IRES-ZsGreen (BEI Resources NR-52516, Manassas, VA, USA) vector; 1.1 μg each of vectors HDM-Hgpm2 (BEI Resources NR-52517, Manassas, VA, USA), pRC-CMV-Rev1b (BEI Resources NR-52519, Manassas, VA, USA), and HDM-tat1b (BEI Resources NR-52518, Manassas, VA, USA); and 1.7 μg of vector pCMV14-3X-Flag-SARS-CoV-2 S (Addgene Cat # 145780, Watertown, MA, USA). For 6-well plates, the following were mixed with 185 μL of serum-free commercial Dulbecco’s Modified Eagle Medium in a sterile 1.5 mL centrifuge tube instead: 1 μg of lentiviral backbone Luciferase-IRES-ZsGreen vector; 0.22 μg each of vectors HDM-Hgpm2, pRC-CMV-Rev1b, and HDM-tat1b; and 0.34 μg of spike vector pCMV14-3X-Flag-SARS-CoV-2 S.

After the DNA vector solutions were mixed by pipetting up and down 10 times, either 30 μL or 8 µL of XtremeGENE HP Version 9 (Roche Cat # 06366546001, Mannheim, Germany) was then added directly to the solution for the 100 mm Petri dishes or 6-well plates, respectively. The tubes of vector transfection mixture were gently tapped about 10 times to mix the solution and were subsequently allowed to incubate at room temperature for 20–25 min. Then, the 100 mm Petri dishes/6-well plates were retrieved, and the DNA + XtremeGENE HP transfection mixture was added dropwise to the HEK 293FT cells. The transfected cells were then returned to the incubator and allowed to recover for 12–18 h.

After 12–18 h, transfected cells were retrieved from the incubator, and old 293 medium was replaced with either 10 mL or 4 mL of fresh 293 medium for 100 mm Petri dishes or 6-well plates, respectively. The transfected cells were returned to the incubator for an additional 48 h to allow for pseudoviral production.

After 48 h had elapsed, the transfected cells were removed from the incubator, and the old 293 medium supernatant (now containing lentivirus) was gently removed and transferred to a 15 mL tube. The lentivirus solution was clarified by centrifugation at 105× *g* for 3 min and then filtered through a 0.45 µm sterile PES syringe filter (ThermoFisher Cat # 09-740-114, Waltham, MA, USA). Filtered lentiviral pseudovirus solution was stored as 450 μL aliquots in low-binding 1.5 mL tubes (ThermoFisher Cat # 90410, Waltham, MA, USA) at −75 °C until use in future assays.

Pseudoviruses with additional structural proteins were exclusively produced in 100 mm Petri dishes. To do so, 1.7 μg of desired M, N, or E protein vector was added to the transfection mixture specified above: pcDNA3.1 SARS-CoV-2 M (Addgene Cat # 158078), pcDNA3.1 SARS-CoV-2 N (Addgene Cat # 158079), and pcDNA3.1 SARS-CoV-2 E (Addgene Cat # 158080), respectively. All other protocols for lentivirus creation and storage were performed unchanged, as described above.

When creating MNE virus/pseudovirus with all three structural proteins incorporated in the virion, 1.7 μg of each additional structural protein vector described above was added to the transfection mixture. When making MNE virus/pseudovirus with all three additional structural proteins, 40 μL of XtremeGENE HP Version 9 was added to the transfection mixture instead of 30 μL. All other protocols for lentivirus creation and storage were performed unchanged, as described above.

### 2.6. Pseudovirus Titration

Lentivirus pseudotyped with Wild-Type SARS-CoV-2 3xFlag spike protein was produced multiple times to ensure results were not due to batch-to-batch variation. Similarly, SARS-CoV-2 pseudovirions with additional structural proteins were produced at least twice to also account for batch-to-batch variation. Every batch of virus was titrated using an XpressBio p24 ELISA kit plate carried out according to the manufacturer’s instructions (XpressBio Cat # XB-1000, Frederick, MD, USA). The ELISA assays were read on a ClarioStar Plus microplate reader using its colorimetric assay feature set to 450 nm (BMG Labtech, Ortenberg, Germany).

### 2.7. Pseudovirus Spike Capture Assay

For virus capture assays, SARS-CoV-2 (2019-nCoV) spike-neutralizing antibody, Rabbit Mab (SinoBiological Inc. Cat # 40592-R001, Beijing, China), was thawed on ice and diluted 2830-fold in ELISA Coating Buffer (70 mM NaHCO_3_, 30 mM Na_2_CO_3_, pH = 9.6) to obtain a 1 µg/mL stock solution. Then, 100 µL of antibody stock solution was added to each well of a Nunc MaxiSorp™ high protein-binding capacity 96-well ELISA plate (ThermoFisher Scientific, Cat # 44-2404-21, Waltham, MA, USA) and was allowed to incubate overnight at 4 °C to allow for the antibody to coat the wells of the plate.

While the ELISA plate was incubating, Blocking Buffer was prepared by adding 3% Bovine Serum Albumin (Sigma Chemical Company, Cat # A-4503, St. Louis, MO, USA) to 50 mL of commercial PBS and allowed to dissolve overnight at ambient temperature.

After overnight incubation, the ELISA plate was retrieved from the 4 °C refrigerator, and each well was rinsed three times with 300 μL of commercial PBS. Wells were then blocked by gently dispensing 300 μL of Blocking Buffer. The Nunc MaxiSorp capture plate was allowed to incubate at 37 °C for 1 h to allow for blocking. During this time, tubes of pseudovirus aliquot were retrieved from −80 °C storage and were allowed to thaw on ice.

The ELISA Nunc MaxiSorp capture plate was then retrieved from the 37 °C incubator, and wells were rinsed three times with 300 μL of commercial PBS. Pseudovirus samples were then diluted tenfold with 293 media and then diluted further as a serial dilution, depending on the titer of each sample. 100 μL of these virus dilutions were dispensed into wells. The plate was then incubated at ambient temperature for 3 to 5 h to allow for virus capture.

After incubation, wells were again washed three times with 300 µL of commercial PBS. Then, 50 μL of PBS with 0.5% Triton X-100 (Sigma Chemical Company Cat # X100, St. Louis, MO, USA) was added to each well and allowed to sit for 5 min at ambient temperature to allow pseudovirions to lyse and release p24. After 5 min, the contents of each well were transferred from the Nunc Maxisorp capture plate to an XpressBio p24 ELISA kit plate (XpressBio Cat # XB-1000, Frederick, MD, USA). Then, 100 μL of PBS was added to each well of the Nunc Maxisorp capture plate, followed by 10 μL of 10× XpressBio Lysis Buffer (provided in HIV-1 p24 ELISA Kit, XpressBio Cat # XB-1000, Frederick, MD, USA) and was allowed to sit at ambient temperature for 10 min to allow for lysis. Contents of each well were once again transferred to the same wells of the XpressBio p24 ELISA kit plate as described previously. Finally, 50 µL of commercial PBS was added to each well of the Nunc Maxisorp capture plate and allowed to sit at room temperature for 5 min. After 5 min, the PBS solution was transferred from each well of the Nunc Maxisorp capture plate to the requisite well of the XpressBio p24 ELISA kit plate as described previously. The XpressBio p24 ELISA kit plate was placed at 37 °C to incubate for an hour.

After 1 h, the XpressBio p24 ELISA kit plate was retrieved from 37 °C, and the p24 ELISA assay was then carried out according to the manufacturer’s instructions as described in Section 2.6 above. As described in Section 2.6, the levels of p24 in each well were measured on a ClarioStar Plus microplate reader set to 450 nm (BMG Labtech, Ortenberg, Germany).

### 2.8. Spike Direct Quantification Assay

To quantify the level of spike protein present in SARS-CoV-2 pseudotyped lentivirus samples, virus aliquots that were titered as described in Section 2.6 were retrieved from −80 °C storage and allowed to thaw on ice. Samples were then diluted in Standard/Sample Diluent (R1) (ABclonal Cat # RM00023, Woburn, MA, USA). We found that virus samples that provided reliable reads at 1:10,000 dilution on the tittering assay in Section 2.6 needed to be diluted 25-to-100-fold to provide reliable results on this assay.

After samples were diluted, each sample of virus was run on a SARS-CoV-2 Spike S1 Protein ELISA Kit according to the manufacturer’s instructions (ABclonal Cat # RK041543, Woburn, MA, USA). The ELISA assays were read on a ClarioStar Plus microplate reader using its colorimetric assay feature set to 450 nm (BMG Labtech, Ortenberg, Germany).

### 2.9. Virus Direct Infectivity Assays of HEK-293 Cells

On day 1, each well of a clear 96-well cell-culture plate was coated with 25 μL of 0.1 mg mL^−1^ poly-L-lysine (ScienCell Research Laboratories, Cat # 0413, Carlsbad, CA, USA). The plate was returned to the 37 °C incubator for 1 to 36 h. After this time, the 96-well plate was removed from the incubator, and the poly-L-lysine solution was pipetted out. Wells were then rinsed twice with 40 μL of sterile, ultrapure deionized water before being set aside in preparation for seeding.

After wells were prepped, 15,000–25,000 of HEK 293T-hACE2+ cells were seeded in triplicate into the wells of a pre-prepared poly-L-lysine-conditioned 96-well plate. As a control, at least three wells were seeded with 15,000–25,000 HEK-293FT cells. The 96-well plate was returned to the 37 °C humidified incubator at 4.5% CO_2_ in order to allow the HEK-293 cells to adhere and recover for 8–12 h.

While cells recovered, Griffithsin proteins were resuspended in sterile commercial Phosphate-buffered Saline (PBS) (ThermoFisher Cat # 14190-136, Waltham, MA, USA) to create a stock solution. Griffithsin protein was serially diluted in at least 80 μL of PBS for the inhibition assay. Alternatively, SARS-CoV-2 (2019-nCoV) spike-neutralizing antibody, Rabbit Mab (SinoBiological Inc. Cat # 40592-R001, Beijing, China), was thawed on ice and diluted 47-fold in PBS to obtain a stock concentration of approximately 399 nM. Antibody was then serially diluted in at least 80 μL of PBS for the inhibition assay.

After cells had recovered, the 96-well plate was retrieved from the incubator, and the media were gently pipetted out of wells. Within 1 min of pipetting out media, 20 μL of fresh 293 media was added to the wells to prevent the cells from desiccating. Then, 20 μL of the requisite Griffithsin or antibody protein dilution in PBS was added in triplicate to wells. Wells were allowed to equilibrate for 10 min, and then 20 μL of SARS-CoV-2 pseudovirus variant was added to the wells. The 96-well plate was subsequently returned to the 37 °C humidified incubator at 4.5% CO_2_ in order to allow the HEK-293 cells to adhere and recover.

Then, 12–16 h later, the 96-well plate was retrieved from the humidified incubator, and 150 μL of fresh, pre-warmed 293 medium was gently dispensed into each well to ensure that cells remained alive for the duration of the experiment. The 96-well plate was then returned to the 37 °C incubator for an additional 36 to 48 h.

After 36 to 48 h had elapsed, the 96-well plate was retrieved from the humidified incubator and prepared for luminescence readings. Luciferase assays were performed as described previously [65]. Briefly, 160–180 μL of the medium in each of the infectivity plate wells was pipetted out, leaving about 30 μL of the medium in each well after accounting for evaporation during the course of the experiment. 30 μL of Bright-Glo Luciferase Reagent (Promega Corp., Cat # E2610, Madison, WI, USA) was added to wells and allowed to lyse cells for 2–4 min. The contents of each well were then transferred to a white-backed 96-well plate, and luciferase signal was read on a ClarioStar Plus microplate reader utilizing the luminescence feature with a 3600 gain and a 1 s normalization time. All samples were run in triplicate with at least two separate biological replicates for each condition.

### 2.10. Virus Direct Infectivity Control Assays of Raji Cells

For direct infectivity controls for Raji cells, 96-well plates were prepped with poly-L-lysine, as described above. Then, 15,000–25,000 cells of HEK 293T-hACE2+ cells in 100 μL and 15,000–25,000 cells of either Raji DC-SIGN+ cells or Raji Wild-Type cells in 100 μL were seeded in triplicate into the wells of a pre-prepared poly-L-lysine-conditioned 96-well plate and allowed to recover for 8–12 h in a 37 °C humidified incubator at 4.5% CO_2_. This allowed HEK 293T-hACE2+ cells to adhere to wells and Raji cells to fall to the bottom of wells.

After 8–12 h, 50 μL of media was gently removed from wells. From our observations, although the Raji cells did not appear to adhere to wells, they did not move from the bottom of the wells when we removed media. Then, 50 μL of SARS-CoV-2 pseudovirus solution was added over the top of the wells, and the 96-well plate was subsequently returned to the 37 °C humidified incubator at 4.5% CO_2_ in order to allow the HEK-293T-hACE2+ cells and Raji cells to undergo infection.

Then, 12–16 h later, the 96-well plate was retrieved from the humidified incubator, and 150 μL of fresh, pre-warmed 293 medium was added over the top of each 293T-hACE2+ well. A total of 150 μL of fresh, pre-warmed Raji medium was added over the top of each Raji DC-SIGN+/Raji Wild-Type well. This was to ensure that cells remained alive and viable for the duration of the experiment. The 96-well plate was returned to the 37 °C humidified incubator for an additional 36 to 48 h.

After 36–48 h, the 96-well plate was retrieved from the humidified incubator, and Bright-Glo Luciferase Reagent luminescence assays were performed as described in Section 2.7 above. Empirically, Raji cells did not appear to move or detach from the wells when media were gently removed from wells.

### 2.11. Virus Raji Cell-Mediated Trans-Infectivity Assays

Raji cell trans-infection assays were inspired by protocols described previously with some modifications [66,67,68,69]. Briefly, for trans-infection experimental samples, 10,000–20,000 HEK-293T hACE2+ cells were seeded in triplicate into the wells of a pre-prepared poly-L-lysine-conditioned 96-well plate, as described above. For negative control samples, HEK-293FT cells were seeded in triplicate into wells instead. The 96-well plate was returned to the 37 °C humidified incubator at 4.5% CO_2_ in order to allow the HEK-293 cells to adhere and recover for 8–16 h overnight.

The next day, Griffithsin protein sample was then resuspended in commercial PBS, as described above, and was serially diluted with PBS so that at least 500 μL of Griffithsin protein was present for each dilution concentration. A total of 400 μL of each Griffithsin dilution was aliquoted into a 1.5 mL low-protein-binding microcentrifuge tube (ThermoFisher Cat # 90410, Waltham, MA, USA). For positive controls, a sample of 400 μL PBS was prepared in one of the 1.5 mL low-protein-binding microcentrifuge tubes instead.

Next, the Raji DC-SIGN+ cell flask and Raji Wild-Type cell flask were retrieved from the 37 °C humidified incubator. About half of the cell media was collected in a 50 mL centrifuge tube and pelleted by centrifugation at 200× *g* for 3 min. Supernatant was carefully removed with a serological pipet, and each Raji cell type was taken to 5–10 mL of 200,000–400,000 cells per mL with fresh Raji media. Then, 250 μL of this cell suspension was added to a 1.5 mL low-protein-binding microcentrifuge tube containing a serial Griffithsin dilution in PBS as specified above.

Finally, 55 μL of SARS-CoV-2 pseudoviral solution was added to each low-protein-binding microcentrifuge tube. The mixture of SARS-CoV-2 pseudovirus, Raji cells, and Griffithsin protein in PBS was allowed to incubate at room temperature for 2 h. After incubation, the cells were centrifuged for 15 min at 100× *g* to gently collect the cells. Supernatant was gently removed, and pellets were resuspended with 200 μL of 2% Fetal Bovine Serum in PBS (heretofore referred to as Washing Buffer). Cells were centrifuged again for 15 min at 100× *g,* and the supernatant was gently removed. Pellets were once again resuspended with 200 μL of Washing Buffer. Samples of cells were collected by centrifuging for 15 min at 100× *g,* and the supernatant was gently removed for a total of two washes. Pellets containing Raji cells with captured pseudovirus were then resuspended in 90 μL of DMEM supplemented with 10% Fetal Bovine Serum (heretofore referred to as Final Infectivity Media).

The 96-well plate with seeded HEK-293 cells was then retrieved from the humidified incubator, and media within wells were gently replaced with 30 μL of fresh 293 media. A total of 30 μL of Raji cells with captured pseudovirus in Final Infectivity Media, as described above, were added to requisite wells. The 96-well plate was then returned to the 37 °C humidified incubator at 4.5% CO_2_ in order to allow the captured pseudovirions to infect the adhered 293 cells.

Then, 12–16 h later, the 96-well plate was retrieved from the humidified incubator, and 90 μL of fresh, pre-warmed 293 medium was added over the top of each well to ensure that cells remained alive and viable for the duration of the experiment. The 96-well plate was returned to the 37 °C incubator for an additional 36 to 48 h.

After 36–48 h, the 96-well plate was retrieved from the humidified incubator, and luminescence activity was measured using a ClarioStar Plus microplate reader as described for direct infectivity assays in Section 2.7 and Section 2.8 above.

When carrying out mannan inhibition of Raji cell capture, a dilution series of mannan in PBS was created instead of a serial Griffithsin dilution. Mannan polymers from Saccharomyces Cerevisiae (Sigma-Aldrich Cat # M7504-100MG, Saint Louis, MO, USA) were utilized for the assay. To prepare the dilutions, 100 mg of mannan was dissolved in 40 mL of ultrapure water to create a 2.5 mg mL^−1^ stock solution of mannan. This 2.5 mg mL^−1^ stock solution was serially diluted with PBS in low-binding tubes for the Raji trans-infectivity assay. The Raji cells were pipetted into the mannan dilutions, and the protocol described in Section 2.9 was continued with no additional changes.

### 2.12. Virus 3t3 Cell-Mediated Trans-Infectivity Assays

Trans-infection assays were performed as described in the previous literature [65]. Briefly, for trans-infection experimental samples, 5000–15,000 of 3t3 DC-SIGN+ cells were seeded in triplicate into the wells of a pre-prepared poly-L-lysine-conditioned 96-well plate as described above. 3t3 Wild-Type cells were seeded into negative control wells for trans-infection assay. The 96-well plate was returned to the 37 °C humidified incubator at 4.5% CO_2_ in order to allow the 3t3 cells to adhere and recover for 8–12 h.

For trans-infectivity tests with Griffithsin inhibitors, two different protocols were tested: Griffithsin pre-incubated with virus and Griffithsin pre-incubated with 3t3 cells.

For the Griffithsin pre-incubated with virus protocol, 10 μL of the desired dilution of the requisite Griffithsin variant (WT-Grft, Q-Grft, or GLG-3A) was added to 30 μL of SARS-CoV-2 pseudotyped lentivirus solution and left at room temperature to incubate. After 20 min of Griffithsin incubation with virus, the 96-well plate was retrieved from the humidified incubator, and the media were gently pipetted out of wells. Within 1 min of pipetting out the media, the 40 μL Griffithsin-pseudovirus mixture was added to the well. Each Griffithsin dilution was run in triplicate.

For the Griffithsin pre-incubated with 3t3 cells protocol, 50 μL stock dilution of each requisite Griffithsin variant (WT-Grft, Q-Grft, GLG-3A) was prepared in PBS. The 96-well plate was then retrieved from the humidified incubator, and the media were gently pipetted out of wells. Within 1 min of pipetting out media, the 10 μL of Griffithsin variant in PBS was added to the requisite well, and the 96-well plate was left at room temperature to incubate for 20 min. We found that 10 μL of PBS was a sufficient volume to ensure enough moisture remained in the wells to prevent 3t3 cells from desiccating during the 20 min incubation time period. After 20 min had elapsed, 30 μL of SARS-CoV-2 pseudotyped lentivirus was added to the wells. For data analysis, the luciferase signals from wells were normalized to the signal from control wells of 3t3 DC-SIGN+ trans-infection without any inhibitor.

For trans-infectivity tests with SARS-CoV-2 pseudotyped lentiviral variants (M, N, E, and MNE trans-infectivity tests), the 96-well plate was retrieved from the incubator after cells had recovered for the allotted 8–12 h. The media were gently pipetted out of wells. Within 1 min of pipetting out media, 15 μL of fresh 3t3 media was added to the wells to prevent the cells from desiccating. Then, 25 μL of the requisite SARS-CoV-2 pseudovirus variant was added over the top of the wells.

After the pseudovirus was added to the wells of the 96-well plate, the plate was returned to the 37 °C humidified incubator at 4.5% CO_2_ for 2–4 h to allow for virion capture.

After 2–4 h had elapsed, the 96-well plate was retrieved from the humidified incubator, and the experimental wells were rinsed twice with 40 μL of fresh 3t3 media exactly as described in previous work [65]. After rinsing, HEK-293T hACE2+ T-75 flasks were passaged so that 15,000 to 25,000 HEK-293T hACE2+ cells in 293 media could be added into each well. Then, the 96-well plate was returned to the incubator to allow for cell recovery and viral trans-infection to occur.

Then, 12–16 h later, the 96-well plate was retrieved from the humidified incubator, and 150 μL of fresh, pre-warmed 293 medium was added over the top of each well as described in Section 2.7, Section 2.8 and Section 2.9 above.

After 36–48 h, the 96-well plate was retrieved from the humidified incubator, and luminescence activity was measured using a ClarioStar Plus microplate reader as described in Section 2.7, Section 2.8 and Section 2.9 above.

For mannan inhibition of 3t3 cell DC-SIGN-mediated trans-infection, mannan polymers from Saccharomyces Cerevisiae were utilized as described in Section 2.9 above. When performing the 3t3 cell-mediated trans-assay, 15 μL of 0.053 mg mL^−1^ mannan solution was added to 3t3 cell wells just prior to adding 25 μL of pseudovirus to obtain 0.02 mg mL^−1^ (20 μg mL^−1^) of mannan, as described in the previous literature [65].

## 3. Results

### 3.1. Wild-Type Griffithsin and M78Q Griffithsin Both Display Moderate Anti-SARS-CoV-2 Inhibitory Capabilities

**Figure 1:**
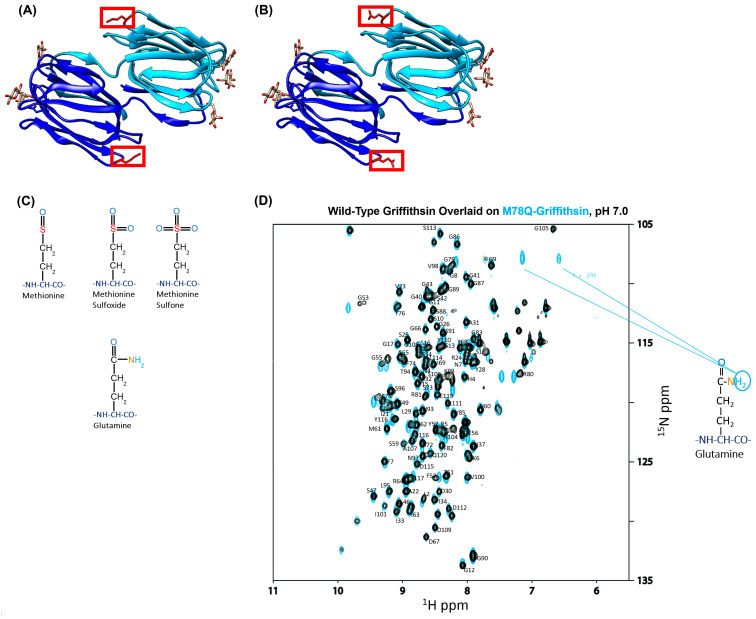
(**A**,**B**) The schematic diagram of Wild-Type Griffithsin compared to M78Q Griffithsin. The location of Methionine 78 is highlighted in red. On the left, (**A**), is a depiction of WT-Grft. On the right, (**B**), is a depiction of Methionine 78 mutated to Glutamine (also known as Q-Grft). Griffithsin structures are derived from PDB ID: 2NUO. (**C**) Depiction of post-translational oxidation that occurs on Methionine. (**D**) NMR spectrum overlay of Q-Griffithsin (teal blue) underneath WT-Griffithsin (black). The overlay shows peaks with a high degree of concordance, indicating that the fold of Q-Grft matches the fold of WT-Grft. The spectrum of WT-Grft was compared to previous spectra of functional Griffithsin produced previously [22,31]. The expected peaks of the sidechain Q78 Glutamine are pointed out in teal.

While Wild-Type Griffithsin has been tested as a potential antiviral against COVID-19, oxidation has been found to occur at Methionine 78 (Figure 1A–C), which complicates its status as a viable therapeutic for use in humans since the Food and Drug Administration requires a thorough assessment of post-translational modifications on protein-based therapeutics [24,29,66,72]. To avoid this, researchers have found that mutating M78 to Glutamine alleviates these issues, making M78Q Griffithsin an easier and more feasible candidate to use for clinical trials [70].

Before embarking on assessing whether Griffithsin could act as a suitable inhibitor of SARS-CoV-2 viral infection, we first verified that the M78Q mutation did not disrupt the native fold of WT-Grft. This was important because although the antiviral/prophylactic capability of recombinant Q-Grft has been verified to match WT-Grft in the literature, there does not yet appear to be published verification that recombinant production of M78Q Griffithsin in E. coli does indeed produce a protein that exhibits structural similarity to WT-Grft [29,40,70,73]. To do so, we produced ^15^N-labeled WT-Grft and Q-Grft (as described in the Materials and Methods Section 2.2 above) and then assessed both proteins by NMR HSQC (Figure 1D). Our spectra show that not only did Q-Grft retain the same overall fold of WT-Grft but also that the Q78 amino acid was clearly discernable on the NMR, as evidenced by the presence of its twin side-chain hydrogens at the same ^15^N ppm value (Figure 1D) [74].

**Figure 2:**
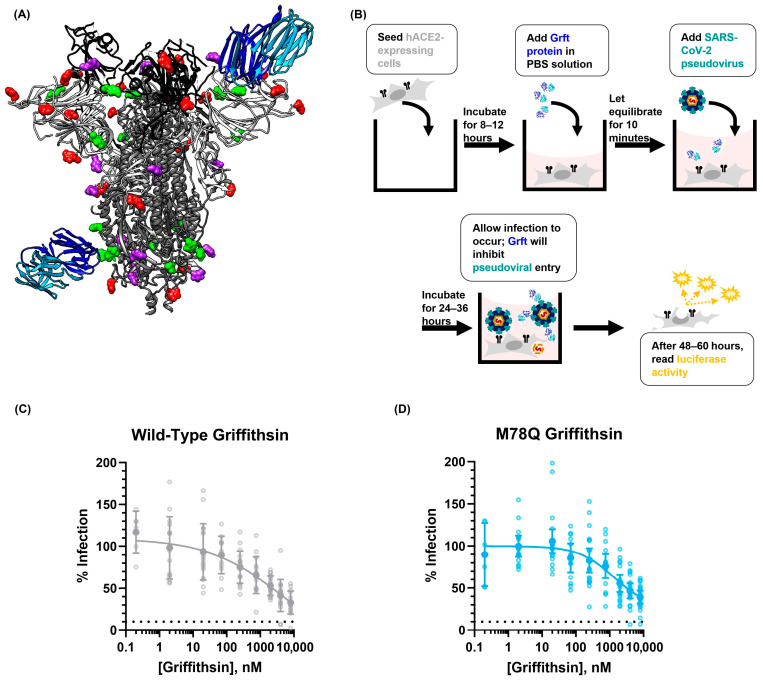
Depiction of how Grft may impede SARS-CoV-2 spike-mediated infectivity. (**A**) Representation of Griffithsin (PDB ID: 2NUO) binding onto the SARS-CoV-2 spike glycans. To depict a high resolution of the loop regions where N-linked glycans are present, two spike PDB structures were overlaid on each other (PDB ID: 7NT9 and 6VYB). The glycans are color-coded to match the type of glycan that is present on the spike protein, as reported in the previous literature (predominantly high-mannose glycans are green, predominantly complex glycans are red, and hybrid glycans/glycans with no consensus between high mannose and complex are depicted in purple). (**B**) Schematic depiction of Griffithsin inhibition of hACE2-mediated SARS-CoV-2 viral direct infection. (**C**) Inhibition of SARS-CoV-2 pseudoviral infection by Wild-Type Griffithsin (gray). (**D**) Inhibition of SARS-CoV-2 pseudoviral infection by M78Q Griffithsin (teal blue). (**C**,**D**) The dashed line indicates one-order-of-magnitude-lower infectivity. Data were fit with a four-parameter variable slope curve (GraphPad Prism).

After performing this quality control check, we then sought to test the antiviral capabilities of both the WT-Grft and Q-Grft proteins on pseudoviral infectivity assays. The SARS-CoV-2 spike protein is known to have 22 highly conserved N-linked glycan sites; an analysis of the existing literature reveals that N61, N234, N709, N717, and N801 are all typically occupied by high-mannose glycans, with N122, N165, N603, N1074, and N1098 also showing a >25% propensity to be occupied by high-mannose or hybrid glycans [65]. Since Griffithsin has a sub-nanomolar affinity to high-mannose sugars, it is likely that the aforementioned glycan sites act as epitopes that allow Griffithsin to bind to the spike protein and prevent viral infection (Figure 2A) [3,22,53]. The inhibitory capabilities of the WT-Grft and Q-Grft proteins were tested on a SARS-CoV-2 hACE2-mediated pseudoviral assay, heretofore referred to as a “Direct Infectivity” assay (Figure 2B). In our hands, WT-Grft and Q-Grft showed a moderate ability to impede pseudoviral infection, with IC_50_ values of 2.16 μM and 4.26 μM, respectively (Figure 2C). While these values are not as potent as the inhibitory capabilities of Griffithsin against other viruses, these values are largely within the range of values expected for SARS-CoV-2 pseudoviral inhibition (Table S1) and are still considered to be potent enough for Griffithsin to be potentially used as a viable agent to develop prophylactic treatments [35].

### 3.2. Removing the Cross-Linking Capability of Griffithsin Does Not Appear to Affect the Inhibitory Capability of Griffithsin

**Figure 3:**
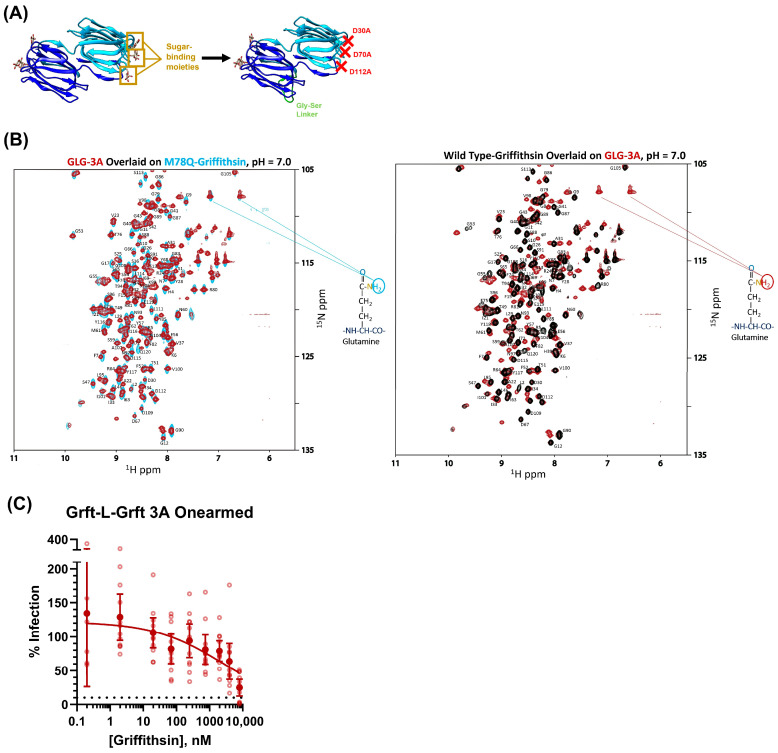
(**A**) Depiction of how the obligate dimer GLG-3A was produced. M78Q Griffithsin monomers were linked with a 16-amino-acid Glycine and Serine linker (depicted in green). The sugar-binding Aspartates on one of the monomers were mutated to Alanines to eliminate the ability of Griffithsin to cross-link proteins. Griffithsin structures are derived from PDB ID: 2NUO. (**B**) The NMR overlay of GLG-3A shows high degree of overlap with both M78Q Griffithsin (left, dark red GLG-3A overlaid onto teal Q-Grft) and Wild-Type Griffithsin (right, black WT-Grft overlaid onto dark red GLG-3A), indicating the protein is folded and functional. GLG-3A has M78Q mutations, which are indicated on the spectra. (**C**) Inhibition of SARS-CoV-2 pseudotyped lentivirus with GLG-3A on the same assay, as depicted in Figure 2B. Dashed line indicates one order of magnitude lower infectivity. Data were fit with a four-parameter variable slope curve (GraphPad Prism).

A key trait of the Griffithsin protein is that it is a domain-swapped dimer with each monomer’s three sugar-binding moieties oriented in opposite directions relative to each other (Figure 3A). This endows Griffithsin with the ability not only to exhibit bivalent binding to a target but also to cross-link target proteins together. This cross-linking capability allows for Griffithsin to aggregate viral spike glycoprotein analogs, such as HIV-1 env protein and Hantaan virus glycoprotein, which is hypothesized to be crucial in maintaining Griffithsin’s inhibitory capabilities against these viruses [26,31,32,75]. In addition, recent work has shown that lectin protein-mediated aggregation of SARS-CoV-2 viral spike proteins is necessary for maintaining the inhibition of authentic SARS-CoV-2 virus and SARS-CoV-2 lentiviral pseudovirus [76]. Despite the fact that both WT-Grft and Q-Grft did not show particularly potent antiviral activity on direct infectivity assays, we still wished to investigate whether this cross-linking capability of Griffithsin was important in the context of inhibiting SARS-CoV-2 spike-mediated infection. Q-Griffithsin is currently in clinical trials as a COVID-19 prophylactic, meaning that even if it does not have exceptional inhibitory capabilities against SARS-CoV-2, it still may be a pharmacologically favorable, non-immunogenic starting point for either a combinatorial prophylactic treatment or for the creation of a better SARS-CoV-2 viral entry inhibitor [28,30]. Hence, uncovering the key structural and functional features governing the inhibitory capabilities of the Griffithsin would assist in altering the protein to produce a better prophylactic against SARS-CoV-2.

We first attempted to abolish bivalent binding by creating Monomeric Griffithsin (Mono-Grft), which has been described previously [26]. Although the Mono-Grft that we produced appeared to be well-folded on NMR, the sample was only able to be resuspended into pH 2.5 buffer, and it aggregated over the course of an hour at pH 6.5–7.0 (Figure S1A,B). The poor solubility of Mono-Grft made it unsuitable for further testing as a potential antiviral therapeutic.

To work around this, we produced a construct inspired by previous work from our group whereby the M78Q Griffithsin dimer is expressed as a single polypeptide chain with a 16-amino-acid-long Glycine–Serine linker between the two Griffithsin monomers [22,31]. The three sugar-binding moieties on one of the Griffithsin monomer domains were removed via mutagenesis, yielding a constitutively dimeric form of Q-Griffithsin with only one monomer domain being able to bind to sugars (Figure 3A). This “Onearmed” variant of Q-Griffithsin retains the overall structure of the Griffithsin dimer but is only capable of monovalent binding to sugar epitopes. This variant (heretofore referred to as GLG-3A) was produced with M78Q mutations on both domains of the Griffithsin dimer, and after its size was verified by SDS-PAGE, its structure was verified to be similar to both WT-Grft and Q-Grft by NMR HSQC (Figure 3B and Figure S2).

When GLG-3A was tested on our direct infectivity assay, as depicted in Figure 2B, it appeared to behave similarly to both WT-Grft and Q-Grft, albeit with an approximately 1-to-3-fold diminished antiviral capability (IC_50_ = 4.89 μM) (Figure 3C). This is in contrast to GLG-3A inhibition tests with HIV-1 pseudovirus, whereby removing the crosslinking ability of Griffithsin diminished its antiviral activity by approximately 100-to-1000-fold [31]. Ergo, while it appears as though the cross-linking and spike aggregation are crucial in mediating the antiviral capability of Griffithsin in the context of HIV, it does not appear to greatly contribute to the etiology of Griffithsin-based SARS-CoV-2 pseudoviral inhibition.

### 3.3. Griffithsin Enhances DC-SIGN-Mediated Trans-Infection in Raji Cells

Although the cells in the human lung tissue only modestly express hACE2, researchers have shown that attachment receptors on the surface of airway epithelial cells and other respiratory system resident cells are likely responsible for capturing SARS-CoV-2 virions and presenting them to cells that slightly express hACE2. Specifically, the lectin receptor DC-SIGN has a proclivity to bind to high-mannose sugars on the surface of viral glycoproteins, allowing it to facilitate efficient infection-in-trans (also called trans-infection) of several viruses, including human T-cell lymphotropic virus type 1, Enterovirus 71, Dengue Virus, HIV-1, and, most recently, SARS-CoV-2 [33,34,53,54,59,60,61,62].

**Figure 4:**
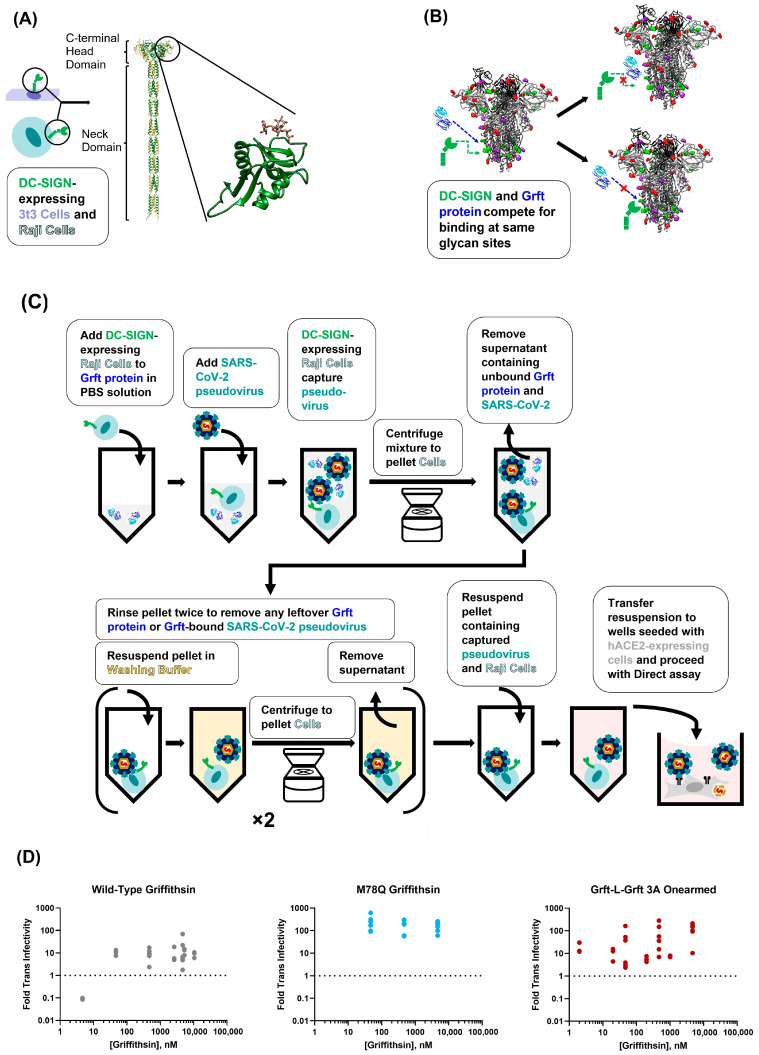
Griffithsin appears to enhance DC-SIGN-mediated trans-infection via Raji cells. (**A**) Simplified depiction of the DC-SIGN lectin receptor on the surface of 3t3 and Raji cells. The DC-SIGN extracellular domain ribbon structure is an AlphaFold prediction [77,78]. The structure was chosen by selecting the closest prediction to a previously constructed structure from Tabarani, G. et al. [79]. We further zoom in on the C-terminal Carbohydrate Recognition Domain (CRD) of DC-SIGN bound to mannan (PDB ID: 1SL4). (**B**) Depiction of how DC-SIGN CRD theoretically competes with Griffithsin for binding to high-mannose N-linked glycans on the SARS-CoV-2 spike protein. The spike protein structure is an overlay of PDB ID: 7NT9 and 6VYB. (**C**) Diagram depicting the process of Raji DC-SIGN+ cell-mediated SARS-CoV-2 pseudovirus trans-infection. (**D**) Results of Griffithsin inhibition of Raji DC-SIGN+ cell-mediated trans-infection. WT-Grft was tested first and was found to consistently enhance trans-infectivity. Q-Grft appeared to behave similarly, although it enhanced trans-infectivity to a greater extent than WT-Grft. GLG-3A was then tested to observe whether Griffithsin-mediated cross-linking could explain the enhanced infectivity of WT-Grft and Q-Grft. GLG-3A also appears to enhance trans-infectivity on the Raji cell-mediated assay. The dashed line indicates the level of trans-infectivity in the absence of any Griffithsin inhibitor.

Because Griffithsin, much like DC-SIGN, is also a lectin that binds to sugars on viral glycoproteins, we theorized that Griffithsin could act as a potent competitive inhibitor of DC-SIGN-mediated trans-infection (Figure 4B) [22]. To test this, we utilized the paired Raji/Raji-DC SIGN+ cell lines that have already been used as a trans-infection model for HIV-1, human T-cell lymphotropic virus type 1, Enterovirus 71, and Ebola Virus [59,60,66,67,68,69]. Our assay was performed similarly to others in the literature, where SARS-CoV-2 pseudovirus and Raji cells were incubated with varying concentrations of inhibitor (in this case, a Griffithsin variant) before Raji cells were washed and added to susceptible hACE2-expressing cells (Figure 4C).

Before utilizing Raji cells as the capture cell line, we first established that they could not be infected with SARS-CoV-2 pseudovirus (Figure S3A,B). We then verified that Raji-DC-SIGN+ cells exhibited higher trans-infectivity than Raji-WT cells. Although Raji-WT cells exhibited only 3-fold worse trans-infection than Raji-DC-SIGN+ cells (indicating that perhaps there were other attachment receptors on the surface of Raji cells that could contribute to viral transfer), the augmented trans-infection signal for Raji-DC-SIGN+ cells was large enough for us to continue with the capture assays (Figure S4). Finally, we confirmed that Raji DC-SIGN+ cell-mediated trans-infection was facilitated via DC-SIGN binding to sugars on the surface of the virion by showing that mannan polymers can act as competitive inhibitors of SARS-CoV-2 virus capture (Figure S5A,B). Taken together, these experiments provided strong evidence that Raji DC-SIGN+ cells were indeed capturing SARS-CoV-2 pseudotyped virions via DC-SIGN protein binding to glycans on the surface of the viral particles.

Using this Raji trans-infectivity assay, we found that 4.7 nM of WT-Grft appeared to inhibit Raji DC-SIGN+ cell-mediated trans-infection (Figure 4D). Oddly, when the concentration of WT-Grft was increased to 47 nM and higher, the SARS-CoV-2 pseudovirus appeared to display greatly enhanced infectivity, reaching infectivity values that were 1 order of magnitude higher than samples without any Griffithsin present.

Our initial hypothesis to explain this phenomenon was that the aforementioned cross-linking capabilities of Griffithsin could tether multiple viruses together, thereby allowing a single DC-SIGN receptor to capture more than one virion [32]. Because the SARS-CoV-2 spike protein has numerous high-mannose glycosylation sites, there conceivably could be enough epitopes on the virion to allow for both Griffithsin and DC-SIGN to bind to the same spike protein. This Griffithsin-mediated cross-linking could, in turn, allow a single DC-SIGN receptor to capture and deliver clusters of multiple SARS-CoV-2 virions provided to susceptible cells. Alternatively, it could be possible that Griffithsin cross-linked SARS-CoV-2 pseudoviral particles to such an extent that the virions were able to be pelleted by centrifugation without the need to be captured by DC-SIGN-expressing cells. To test these hypotheses, we performed our experiments with the monovalent variant of Griffithsin that was incapable of cross-linking proteins together (GLG-3A). Contrary to expectations, we observed a similar phenomenon as described for WT-Grft: GLG-3A consistently appeared to enhance Raji DC-SIGN+ SARS-CoV-2 pseudoviral infectivity by at least 1 order of magnitude (Figure 4D).

For the purposes of completion, we performed a less intensive screen of Q-Grft to verify that the M78Q mutation did not alter the trend of Griffithsin increasing Raji DC-SIGN+ infectivity. As shown in Figure 4D, 47 nM of Q-Grft was also sufficient to enhance infectivity on our Raji trans-infectivity assay by at least 1 order of magnitude, indicating that Q-Grft, much like WT-Grft and GLG-3A, enhanced Raji cell-mediated trans-infection.

As a B-cell line, Raji cells express a variety of cell surface proteins—such as Toll-like receptors, B-cell receptors, and complement receptors—which can recognize antigens from various pathogenic particles, act as attachment receptors for viruses like HIV-1 or Epstein–Barr Virus, trigger B-cell activation and differentiation, and cause the secretion of a variety of cytokines and cytotoxic molecules [80,81,82,83,84,85]. Due to the dynamic nature of Raji cells, we believed that it was necessary to verify whether Griffithsin could consistently enhance SARS-CoV-2 trans-infection on another DC-SIGN cell assay system [67,68,69].

### 3.4. Griffithsin Has No Effect on DC-SIGN-Mediated Trans-Infection in 3t3 Cells

**Figure 5:**
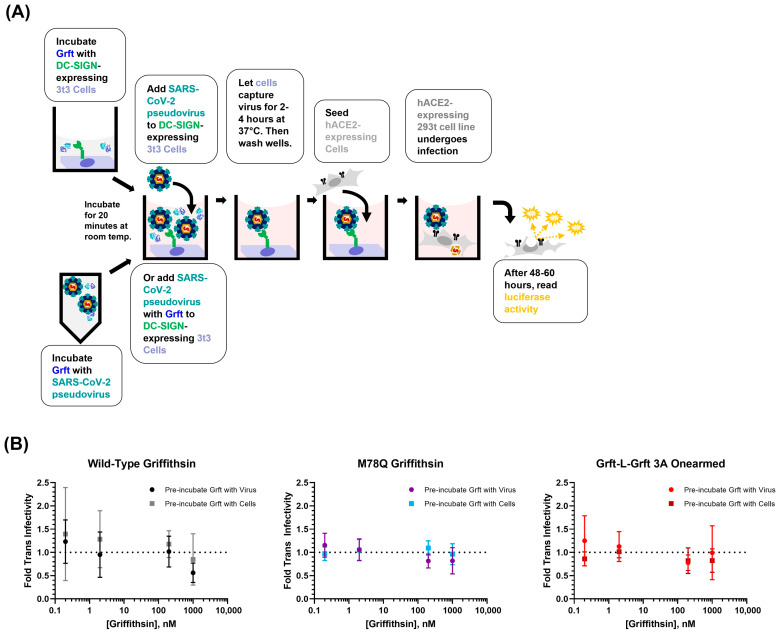
Griffithsin does not affect DC-SIGN-mediated trans-infection in 3t3 cells. (**A**) Diagram depicting the process of 3t3 DC-SIGN+ cell-mediated SARS-CoV-2 pseudovirus trans-infection. Two protocols were tested, where Griffithsin was either pre-incubated with 3t3 cells or with SARS-CoV-2 pseudovirions. (**B**) Griffithsin inhibition of 3t3 DC-SIGN+ cell-mediated trans-infection. Briefly, data points depict 3t3 DC-SIGN+ cell-mediated trans-infection performed in the presence of indicated concentrations of Griffithsin that were then normalized to the signal from 3t3 DC-SIGN+ cell-mediated trans-infection performed in the absence of any inhibitor. Circular data points indicate protocol where Griffithsin was pre-incubated with SARS-CoV-2 pseudovirus before being added to 3t3 DC-SIGN+ cells. Rectangular data points indicate protocol where Griffithsin was pre-incubated with 3t3 cells before SARS-CoV-2 pseudovirus was added to wells. Dotted lines indicate no change in the trans-infectivity signal when compared to control samples without Griffithsin. For both infectivity protocols, none of the Griffithsin variants appeared to have any significant effect on enhancing or inhibiting 3t3 DC-SIGN+ cell-mediated trans-infectivity.

The 3t3 cell line is an adherent murine fibroblast cell line that has low innate expression of lectin receptors or complement receptors and has been shown to not be permissible to SARS-CoV-2 pseudovirus infection [64,65]. We performed 3t3 cell-mediated trans-infection assays as reported previously, whereby pseudotyped lentivirions were captured by adherent 3t3 cells expressing DC-SIGN [65]. To test whether the increase in SARS-CoV-2 pseudoviral trans-infectivity was due to Griffithsin interacting with virions or Griffithsin interacting with DC-SIGN, we performed two different procedures: (1) SARS-CoV-2 pseudotyped lentivirions were pre-incubated with Griffithsin proteins before being adding to 3t3 cells, or (2) Griffithsin was pre-incubated with 3t3 cells before SARS-CoV-2 pseudotyped lentivirus sample was added (Figure 5A). Wells were then washed twice, and susceptible hACE2+ HEK-293T cells were added to wells to allow for infection. After 48–60 h, viral infectivity was measured by luciferase activity assay as described in Figure 2B above.

The results of the DC-SIGN+ 3t3 cell-mediated assay showed that all three Griffithsin variants (WT-Grft, Q-Grft, and GLG-3A) appeared to have no effect on DC-SIGN-mediated trans-infection of SARS-CoV-2 pseudovirus (Figure 5B). Furthermore, neither pre-incubation of Griffithsin with DC-SIGN-expressing cells nor pre-incubation of Griffithsin with SARS-CoV-2 pseudovirus led to a significant difference in viral infectivity. The lack of Griffithsin-mediated enhancement of trans-infectivity on 3t3 cells led us to believe that the enhanced trans-infectivity observed in Section 3.3 with Raji cells (Figure 4D) was likely due to unforeseen signaling changes or interactions in the Raji cell line.

Given the results from the Raji cell and 3t3 cell trans-infectivity assays, we were left to conclude that, at the very least, no variant of Griffithsin (WT-Grft, Q-Grft, and GLG-3A) exhibited any appreciable ability to act as an inhibitor of DC-SIGN-mediated capture of SARS-CoV-2 pseudotyped lentivirions.

### 3.5. Additional SARS-CoV-2 Structural Proteins Do Not Significantly Contribute to Trans-Infection

**Figure 6:**
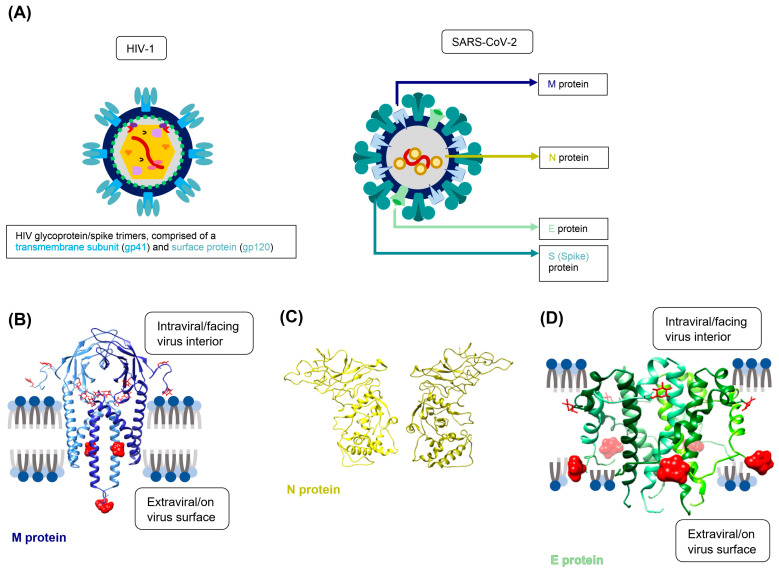
Structure and glycosylation of the three SARS-CoV-2 structural proteins. (**A**) Simplified depiction of SARS-CoV-2 lentiviral pseudovirions (left) versus SARS-CoV-2 coronavirus virions (right). HIV lentivirions incorporate numerous features into their viral particles, as they must carry the machinery necessary to reverse-transcribe the lentiviral RNA genome and incorporate it into the host DNA genome [41]. In contrast, coronaviral particles only incorporate the structural proteins M, N, and E, in addition to the spike protein. Spike, M, and E proteins are present on the surface of coronavirus virions, while the surfaces of lentiviral pseudovirions are essentially bare of any protein aside from spike glycoprotein. (**B**) M protein dimeric structure, with 8 potential N-linked glycosylation sites shown in red. The M protein is the most highly expressed protein on the surface of the SARS-CoV-2 virion and exists as a homodimer. The 2 glycans that are on the extraviral side of the virion and are potentially accessible for DC-SIGN lectin receptor recognition are depicted as space-filling models [86]. The glycan at N216 is on a superimposed loop to complete the structure of the database (PDB ID: 7VGR). (**C**) The N protein exists as a homodimer and is localized to the interior of the virion. It is not anticipated to undergo glycosylation. Rather, it mediates viral RNA genome packaging and regulates host immune responses (PDB ID: 8FG2) [43]. (**D**) SWISS-Model predicted structure of the SARS-CoV-2 E-protein [88,89,90,91,92,93,94,95,96]. The E protein exists as a homopentamer on the viral surface and has two expected glycan sites, depicted in red. Glycan N66 is closer to the extraviral side of the viral membrane and is thus depicted as a space-filling model in the figure [97].

Although HIV lentivirions and SARS-CoV-2 virions share many similar features, they do differ in the number and type of proteins that are incorporated into the final virion [41,42]. HIV lentivirions only express the gp120/41 spike glycoprotein trimers on their surface, with numerous other proteins sequestered inside of the viral particle (Figure 6A). In contrast, the SARS-CoV-2 virus packages only three additional structural proteins within and on the surface of the mature virion. These include (1) the nucleocapsid protein, N, which interacts with the viral genome; (2) the envelope protein, E, which is expressed on the surface of the virions and is important for maintaining viral pathogenesis; and (3) the membrane protein, M, which is also expressed on the virus surface and can potentially act as an ion channel or a sugar transporter (Figure 6B–D) [86,87]. As in other coronaviruses, all three structural proteins interact with each other and the spike to facilitate viral budding, protein transport through the Endoplasmic Reticulum, and viral attachment [46,47,48,49,50].

To our knowledge, there is currently no research that has assessed whether the SARS-CoV-2 structural proteins alter DC-SIGN-mediated trans-infection [46,47]. This is particularly important for adequately modeling the initiation of COVID-19 infection since SARS-CoV-2 is theorized to rely on attachment and presentation by surface proteins like C-type lectin receptors in order to efficiently infect the low-hACE2-expressing cells of the oral epithelium and respiratory airways [52,56]. We gave particular focus to the SARS-CoV-2 M and E proteins, as they are both glycosylated proteins on the viral surface and can, therefore, potentially contribute significantly to SARS-CoV-2 recognition by lectin receptors [48]. The M and E proteins are predicted to have eight glycosites and two glycosites, respectively, although only a subset of these glycans is oriented toward the exterior of the viral particle (Figure 6B,D). Regardless, the presence of both the M and E proteins on the surface of the coronaviral virion could provide a significant number of accessible high-mannose glycans for virus recognition, capture, and trans-infection [86].

**Figure 7:**
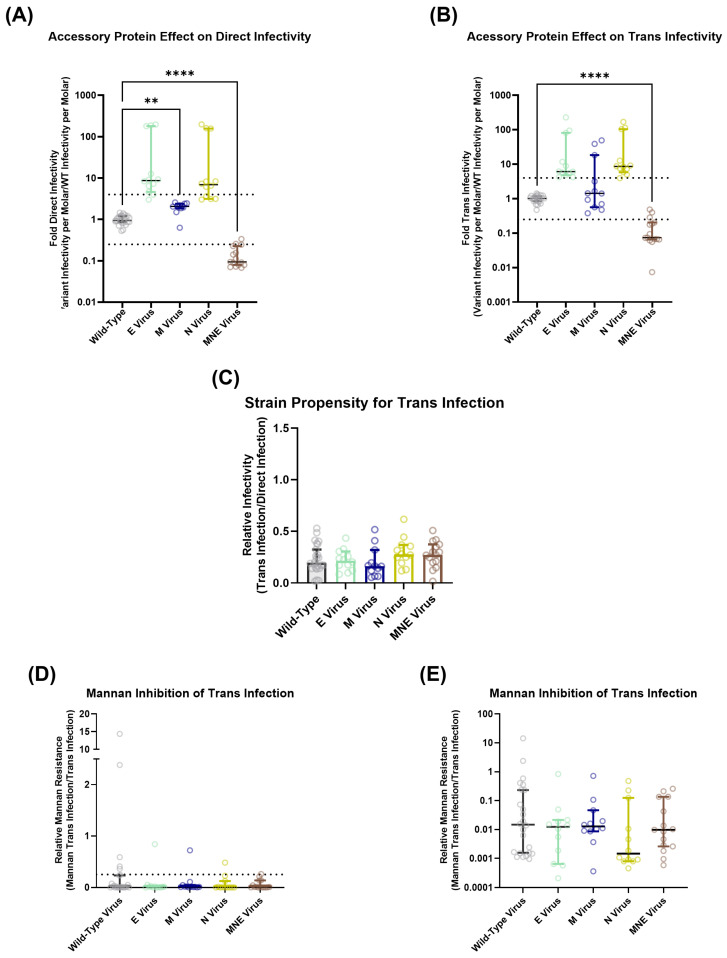
Structural proteins enhance the ability of SARS-CoV-2 pseudovirus to undergo direct infection but have no effect on trans-infection. (**A**) Direct infectivity per molar for each SARS-CoV-2 pseudovirus strain. (**B**) Trans-infectivity per molar for each SARS-CoV-2 pseudoviral strain. (**C**) Each strain’s trans-infectivity signal is normalized to their direct infectivity to deconvolute each strain’s ability to be bound by DC-SIGN from their ability to undergo hACE2-mediated infection. As described in our previous work, this is called the strain propensity to undergo trans-infection [65]. (**D**,**E**) To verify that the viral strains were indeed undergoing DC-SIGN-mediated trans-infection, experiments were performed in the presence of 20 μg/mL of mannan. Mannan decreased trans-infectivity by the same extent for all strains (about 93% inhibition), indicating that the additional SARS-CoV-2 structural proteins do not significantly contribute to DC-SIGN-mediated trans-infection. For ease of visualization, (**E**) depicts the same data with logarithmic values on the *Y*-axis. As with above, dashed line indicates 4-fold decrease in infectivity as inspired by previous literature [98]. (**A**–**E**). Statistical analysis is a Welch ANOVA with an Alpha value of 0.05. ** indicates a *p*-value < 0.01. **** indicates a *p*-value < 0.0001.

To ascertain the effect of the M, N, and E proteins on DC-SIGN-mediated trans-infection, four viral strains were produced: three had one additional structural protein incorporated in the SARS-CoV-2 nCoV-19 Wuhan Wild-Type lentiviral particle (either M, N, or E protein) (Figure S6A,B), and a final strain was produced with all three structural proteins included with the lentiviral system (Figure S6C). As would be expected from the previous literature, the addition of these proteins individually appeared to increase hACE2-mediated direct infectivity for the pseudovirus (Figure 7A) [47]. The per molar infectivity of the MNE strain appeared lower than the Wild-Type spike-only strain despite having a much higher raw luciferase signal than the Wild-Type, E, and M strains (Figure 7A and Figure S7A,C). However, the MNE virus concentration could be artificially inflated by containing a high number of SARS-CoV-2 coronavirus-like particles (cVLPs). Even though these cVLPs should not have the ability to package the luciferase transfer sequence, a significant number of the viral particles likely incorporated some p24 protein due to high expression from the lentiviral backbone vector [46]. This, thereby, likely artificially increased the molarity of MNE virions as calculated by the p24 ELISA immunoassay (Figures S6A,B, S7C, and S8A–D). However, our spike capture and direct spike ELISA assays indicated that the expression of M, N, and E proteins by themselves marginally decreased the average spike expression levels compared to the Wild-Type pseudoviral strain (Figure S8B,D). On the other hand, expression of all three structural proteins appeared to decrease the average number of spike proteins present per virion, although this is likely confounded by the presence of cVLPs artificially inflating the number of viral particles that were measured during virus titration. When we look solely at the raw spike ELISA signal, it appears as though the M, N, and MNE strains have higher spike incorporation per virion when compared to Wild-Type virus (Figure S8A,C).

Running the additional structural protein viral strains on the 3t3 DC-SIGN+ cell-mediated trans-infection assay and normalizing each strain’s per molar luciferase signal (Figure 7B) to their respective hACE2-mediated direct infectivity per molar luciferase signal (Figure 7A) revealed no significant difference between the strains’ propensity to undergo trans-infection (Figure 7C and Figure S9), indicating that there is relatively little interaction between DC-SIGN and the other SARS-CoV-2 surface glycoproteins. A total of 20 μg mL^−1^ of mannan also appears to efficiently prevent all strains’ trans-infection, thereby demonstrating that the presence of additional structural proteins does not impair DC-SIGN’s ability to bind to viral surface glycoproteins and facilitate trans-infection (Figure 7D,E and Figure S10).

### 3.6. Additional SARS-CoV-2 Structural Proteins Modulate Viral Susceptibility to Griffithsin Inhibition of hACE2 Direct Infection

Based on the recent literature, it appears that Griffithsin exhibits more potent inhibitory capabilities against SARS-CoV-2 coronaviral virions than SARS-CoV-2 pseudotyped lentiviral virions (Table S1) [35,36,37,38,39].

Since Griffithsin inhibition is typically mediated via its binding to glycan sites on viral surface proteins, and since SARS-CoV-2 coronaviral particles express more glycoproteins on the surface than lentiviral virions (including M and E structural proteins in addition to viral spike protein), we hypothesized that the additional structural proteins could be responsible for the increased vulnerability of the genuine SARS-CoV-2 coronaviral virions to Griffithsin-mediated inhibition. To test this, we utilized the strains of SARS-CoV-2 pseudotyped lentiviral virions that incorporated M, N, E, or MNE proteins as described in Section 3.4 (Figure S6). Because nearly all of the previous literature used exclusively WT-Grft, and because WT-Grft produced less variability on our direct infectivity assays (see Figure 2C), we exclusively used WT-Grft to assess the effect the additional structural proteins had on the SARS-CoV-2 hACE2-mediated direct infectivity assay, as described in Figure 2B.

**Figure 8:**
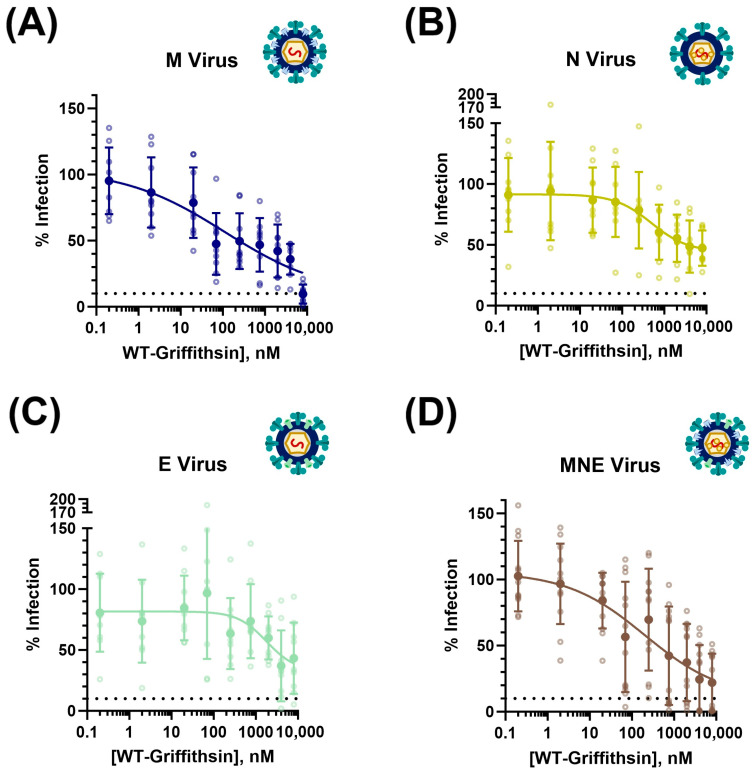
Structural proteins perform variable roles in enhancing and diminishing the ability of SARS-CoV-2 pseudovirus to be inhibited by Wild-Type Griffithsin. (**A**) Inhibition of direct infectivity of SARS-CoV-2 pseudovirus strain expressing M protein shows enhanced susceptibility to WT-Grft; IC_50_ = 0.24 μM. (**B**) Inhibition of direct infectivity of SARS-CoV-2 pseudovirus strain expressing N protein shows diminished susceptibility to WT-Grft; IC_50_ = 3.85 μM. (**C**) Inhibition of direct infectivity of SARS-CoV-2 pseudovirus strain expressing E protein shows diminished susceptibility to WT-Grft; IC_50_ = 2.91 μM. (**D**) Inhibition of direct infectivity of SARS-CoV-2 pseudovirus strain expressing all three additional structural proteins (M, N, and E) shows enhanced susceptibility to WT-Grft; IC_50_ = 0.41 μM. Data were fit with a four-parameter variable slope curve (GraphPad Prism).

Our results indicated that SARS-CoV-2 pseudovirus incorporating the N and E proteins did not display an increased susceptibility to WT-Grft; rather, it seemed as though the N and E proteins increased viral resistance to Griffithsin, with IC_50_ values of 3.85 μM and 2.91 μM, respectively (Figure 8B,C). This is not unexpected, as the addition of both N and E proteins appeared to enhance SARS-CoV-2 pseudoviral infectivity (Figure 7), which could, in turn, allow the virions to retain high infectivity in the face of Griffithsin inhibition [47]. Moreover, N is not expressed on the viral surface, and E protein is only sparsely expressed on the surface of coronaviral particles, meaning that the addition of these structural proteins did not confer a significant number of glycan epitopes for Griffithsin to bind to [99,100].

Meanwhile, both pseudovirus variants that incorporated M protein in the virion (i.e., pseudovirus with only M protein as the additional structural protein as well as pseudovirus with all three (M, N, and E) structural proteins incorporated into the pseudovirus) displayed significantly greater susceptibility to WT-Grft inhibition, with IC_50_ values of 0.24 μM (240.2 nM) and 0.41 μM (412.4 nM), respectively (Figure 8A,D). These results seem to indicate that the M protein is capable of being recognized by Griffithsin, thereby allowing for more potent inhibition. These results largely followed our hypothesis and expectations: given that M protein is the most numerous protein on the surface of the SARS-CoV-2 virion, and it contains multiple accessible putative glycan sites, it is not unexpected that the inclusion of M protein on pseudoviral particles provides multiple epitopes that allow for Griffithsin-based recognition and inhibition [86].

## 4. Discussion

The rollout of COVID-19 vaccines has led to the abatement of SARS-CoV-2-associated mortality [1,2]. However, recent strains of the SARS-CoV-2 virus show a remarkable ability to evade immune detection and breakthrough vaccine inhibition; Omicron sublineages have been shown to engender symptomatic infection in patients who had received up to four vaccine doses [2]. Thus, it is estimated that COVID-19 will persist as an annual disease, much like the common cold or the seasonal flu [2,3]. In fact, the summer of 2023 heralded a surge in COVID-19 infections, coupled with a steady increase in SARS-CoV-2 samples in wastewater [101]. Due to the threat of even mild forms of COVID-19 leading to breakthrough infections that could eventually yield fatal respiratory dysfunction, there is still a need to explore broad-spectrum prophylactic options to combat SARS-CoV-2 entry and infection [2,3,101].

The red-algae *Griffithsia* sp.-derived lectin Griffithsin is well-suited to act as a viral inhibitor: Griffithsin is remarkably stable, is easy to produce in large quantities, is able to be formulated in various materials ranging from liquids to hydrogels to lyophilized powder, is nonimmunogenic, and displays broad antiviral activity [24,25,26,27,101,102]. However, because it can undergo spontaneous oxidation at Methionine 78, it is typical to mutate M78 to Glutamine in order to create a shelf-stable therapeutic that is compliant with FDA bylaws [24,103]. We provide proof in this paper that recombinant Q-Grft can be produced in *E. coli* and does indeed adopt the same overall fold as WT-Grft, as assessed by NMR HSQC. We found that both WT-Grft and Q-Grft moderately inhibit SARS-CoV-2 pseudovirus direct infection in vitro, with IC_50_ values of 2.16 μM and 4.26 μM, respectively.

Another key feature of Griffithsin is that it is a domain-swapped dimer with three carbohydrate binding sites on each monomer, which grants Griffithsin the ability to cross-link viral spike proteins. This cross-linking activity is thought to be crucial in mediating Griffithsin’s antiviral potency against HIV-1 pseudovirus infection in vitro [26,31,32]. To test whether this phenomenon held true for SARS-CoV-2 pseudovirus inhibition, we produced a variant of Griffithsin that was incapable of cross-linking viral proteins (GLG-3A). In our hands, GLG-3A displayed an IC_50_ of 4.89 μM, which is only approximately 2.26- and 1.15-fold higher than WT- and Q-Grft’s IC_50_, respectively, indicating that the cross-linking capability of Griffithsin does not greatly contribute to its inhibitory activity against SARS-CoV-2 pseudotyped lentivirus’ hACE2-mediated direct infection.

We also found that none of these three Griffithsin variants showed any sign of being able to fully attenuate SARS-CoV-2 pseudotyped lentiviral infection; rather, we found that IC_50_ curves plateaued at about 35% to 40% of the maximum signal. Given that we expect our viral samples to, at best, have a concentration of about 0.69 fM, this leads to an estimated 9.66 fM spike proteins that can act as epitopes for Griffithsin binding. Since our Griffithsin concentrations were at least 5 orders of magnitude higher than the spike concentration, it is highly likely that at nanomolar concentrations, Griffithsin was occupying all potentially available epitopes on the surface of the SARS-CoV-2 pseudotyped virions. At the current time, our best hypothesis is that even when Griffithsin fully saturates binding to all available epitopes of the SARS-CoV-2 spike protein, Griffithsin does not bind at locations that totally sterically hinder or impair hACE2 recognition of the spike receptor-binding motif. This hypothesis is somewhat supported by a recent publication where the authors found that Griffithsin does not effectively prevent SARS-CoV-2 spike pseudotyped lentivirus infectivity [76]. We further demonstrated that ~2 nanomolar concentrations of neutralizing antibody were easily capable of preventing SARS-CoV-2 pseuodviral infection, suggesting that the inability of Griffithsin to fully inhibit viral infection was not due to an artifact or other issue relating to excess MOI of our assay (Figure S1). In the future, we hope to perform more experiments to elucidate the binding dynamics and epitope locations involved in Griffithsin binding to spike protein.

During the course of the COVID-19 pandemic, it became apparent that C-type lectin receptors such as DC-SIGN were crucial in mediating SARS-CoV-2 viral infection of cells in the respiratory airways that only modestly express hACE2 [53,54,55,56,57]. Since DC-SIGN exhibits a high affinity for the same high-mannose glycans that Griffithsin binds to, we hypothesized that Griffithsin could be a viable inhibitor of DC-SIGN-mediated trans-infection. To test this, we developed two different trans-infectivity procedures inspired by the previous literature [65,68,69]. When the B-cell lymphoma-derived DC-SIGN+ Raji cell line was used as the virus capture cell line, we found that all three variants of Griffithsin significantly increased DC-SIGN-mediated trans-infectivity.

Since Raji cells are derived from B cells, which are active immune cells, they could be capable of binding to viral pathogen epitopes via Toll-like receptors, B-cell receptors, or complement receptors and undergoing activation [81,82,83,104]. Raji cells specifically express an excess of cell surface receptors when compared to standard B cells, indicating that there is a distinct possibility that they express enough surface receptors to enable T-cell-independent antigen responses [82,84,85]. B cells are also activated by lectins that bind to fucose, leading to cell differentiation and the secretion of proteases and both pro- and anti-inflammatory molecules [105,106]. Finally, Raji cells have recently been discovered to undergo phagocytosis of pathogens, a function that flies in the face of previously established conventions regarding the role of B cells [107]. Because DC-SIGN is a lectin that is capable of binding to fucose, the trans-infection assay process could activate the Raji cells and thereby impede SARS-CoV-2 pseudoviral entry into hACE2+ HEK-293T cells [81,82]. Alternatively, Griffithsin could protect virions from proteases secreted by, or from being phagocytosed by, activated Raji cells, making it appear as though the SARS-CoV-2 pseudovirus infectivity is enhanced by the presence of Griffithsin. In short, B cells engage in numerous different functions and roles within the mammalian body, and so there are many confounding variables that could explain why Raji cells—as a B-cell-derived cell line—could undergo Griffithsin-enhanced trans-infectivity of SARS-CoV-2 pseudotyped virions [108]. All of this served as strong justification for us to move from performing the Raji cell trans-assay to the 3t3 cell trans-assay.

We performed the 3t3 cell trans-assay in a manner inspired by recently published work in our lab [65]. However, to ascertain whether Griffithsin interaction with SARS-CoV-2 pseudotyped virions or Griffithsin interaction with DC-SIGN was responsible for enhancing trans-infection, we performed the assays after pre-incubating the Griffithsin variants with either the 3t3 DC-SIGN+ cells or with virus samples. In the end, it appeared that pre-incubating all variants of Griffithsin (WT-Grft, Q-Grft, and GLG-3A) with virus or with DC-SIGN+ cells had no effect on the ability of SARS-CoV-2 pseudotyped virions to undergo trans-infection.

When the data from both kinds of trans-assays were taken together, the evidence suggests that, at the very least, Griffithsin does not act as a viable inhibitor of SARS-CoV-2 DC-SIGN-mediated trans-infection. Since there are 22 putative glycan sites on each monomer of the SARS-CoV-2 spike protein, it is highly likely that even if Griffithsin blocks DC-SIGN from binding a particular glycan, DC-SIGN can easily shift from binding one glycan to binding to another [56]. Also, Griffithsin does not have a high affinity for fucose, meaning that DC-SIGN could still facilitate trans-infection via binding to fucosylated glycans [23,105,106].

Unlike lentiviral pseudovirions, which only express the SARS-CoV-2 spike protein on their surface, SARS-CoV-2 coronaviral particles also incorporate the M, N, and E structural proteins into viral particles. Of these, M and E are glycosylated membrane-embedded proteins, while the N protein is known to assist with coronaviral genome packaging and is localized to the virus interior [43,46,47,49,50,86]. We wished to assess how these structural proteins affected the infectivity of SARS-CoV-2 pseudoviral particles, with particular focus on whether the structural proteins could enhance the trans-infectivity of SARS-CoV-2 pseudovirions. We were keenly interested in how the M protein affected DC-SIGN-mediated trans-infectivity because, based on data from three other coronaviruses (SARS-CoV-1, Mouse Hepatitis virus, and Feline coronavirus), it is estimated that the SARS-CoV-2 particle expresses ~1100 M protein dimers on its surface [43,86,109]. This leads us to estimate that there are about 2.56-fold more N-linked glycans from the M protein than from the spike protein, meaning that the presence of the M protein could significantly increase the ability of DC-SIGN to bind to the virus particle and facilitate infection.

While the incorporation of the N and E proteins into the SARS-CoV-2 pseudivirion appeared to increase direct infectivity, the propensity for the virus to undergo trans-infection remained largely unchanged (Figure 7C). This was not surprising since the N protein is localized to the interior of the virion; it is, thus, unable to be recognized by cell surface receptors; similarly, the E protein is poorly expressed and incorporated on the surface of viral particles, meaning that it would not provide a drastic increase in glycan epitopes on the surface of the virion [43,97,99,100,110]. Unexpectedly, the presence of the M protein did not appear to enhance the propensity of pseudotyped virions to undergo DC-SIGN-mediated trans-infection at all (Figure 7C). Even when all three additional structural proteins were expressed together, the MNE strain pseudotyped virus displayed no difference in its propensity to undergo DC-SIGN-mediated trans-infection.

We also noticed a consistent trend in the literature whereby Griffithsin seemed to exhibit better inhibitory capabilities against genuine SARS-CoV-2 coronaviral particles than SARS-CoV-2 pseudotyped lentivirions (Table S1). Since structurally, the only difference between the exterior of coronaviral particles and lentiviral particles is the presence of the M and E structural proteins, we hypothesized that the presence of either M or E protein could make viral particles more susceptible to Griffithsin-mediated inhibition of SARS-CoV-2 infection. Indeed, we found that when the M protein was co-expressed and likely incorporated into the SARS-CoV-2 pseudoviral virion, the IC_50_ of WT-Grft on our direct infectivity assays decreased by about an order of magnitude, strongly suggesting that Griffithsin inhibits genuine SARS-CoV-2 coronaviral infection by binding to the M structural proteins on the virion in addition to the spike protein (Figure 8A,D).

Explanations for why Griffithsin binding to the M protein inhibits infection currently elude us; it could also be possible that the M protein interacts with the spike protein to induce it to adopt a conformation that reveals spike glycans in such a manner that they are more easily inhibited by Griffithsin. Alternatively, the binding of Griffithsin to the M protein could sterically hinder the SARS-CoV-2 spike-hACE2 interaction necessary for viral entry and infection. To elucidate the etiology of Griffithsin inhibition, in future work, we believe it would be of interest to quantify the level of the M protein on the surface of the pseudotyped lentiviral virion and to quantify how tightly Griffithsin can bind to the M protein itself.

Taken together, we posit that while Griffithsin favors binding to the M protein, DC-SIGN instead primarily interacts with the glycans on the spike protein. For future studies, we aim to assess whether Griffithsin has any ability to prevent viral trans-infectivity by other attachment receptors that bind to sugar moieties (such as SIGLEC1 and L-SIGN) [54]. Furthermore, we believe it is important to perform more biophysical assessments to verify whether Griffithsin truly has a high affinity for the additional SARS-CoV-2 structural proteins, especially the M protein. Doing this would assist in the creation of an effective Griffithsin-based prophylactic treatment against future strains of COVID-19.

## 5. Conclusions

In short, our results provide some insight into how the antiviral lectin Griffithsin could act as a prophylactic treatment against COVID-19. We found that a variant of Griffithsin that was resistant to oxidation (Q-Grft) displayed similar inhibition of hACE2-mediated SARS-CoV-2 pseudoviral direct infection as Wild-Type Griffithsin. We also found that mutating Griffithsin to abolish its bivalent binding capability (GLG-3A) led to attenuated antiviral activity on the same pseudoviral direct infectivity assays (Figure 3). However, this attenuation was mild when compared with Griffithsin’s prophylactic activity against other viruses, indicating that Griffithsin might dictate its prophylactic activity against SARS-CoV-2 via disrupting a process other than viral spike interaction with the hACE2 receptor [31,32].

We then found that Griffithsin displayed no inhibitory activity against DC-SIGN-mediated viral capture on two different in vitro trans-infection assays (Figure 4 and Figure 5). This suggested to us that even though both DC-SIGN and Griffithsin are lectins that preferentially bind to high-mannose sugars, they do not appear to compete for the same epitopes on the SARS-CoV-2 virus. Given the fact that genuine SARS-CoV-2 coronaviral virions (which incorporate the M, N, and E structural proteins) consistently display greater susceptibility to Griffithsin inhibition than SARS-CoV-2 pseudotyped lentivirions, we hypothesized that Griffithsin mediates COVID-19 inhibition via binding to one of the structural proteins in addition to the SARS-CoV-2 spike protein (Table S1, Figure 6). As predicted, the addition of M, N, E, or MNE proteins into SARS-CoV-2 pseudotyped lentivirions did not alter the DC-SIGN-mediated trans-infectivity of the viral samples, but the addition of the M protein increased the virus’s susceptibility to Griffithsin inhibition. Ergo, this indicated that DC-SIGN facilitates trans-infection by binding to the spike protein, while Griffithsin likely impedes viral infection via binding to the SARS-CoV-2 M structural protein (Figure 7 and Figure 8).

## Data Availability

Data are contained within the article and Supplementary Materials.

## Supplementary Materials

The following supporting information can be downloaded at https://www.mdpi.com/article/10.3390/v15122452/s1. Table S1: A summary of Wild-Type Grifithsin’s antiviral capabilities against SARS-CoV-2 pseudotyped lentivirus versus coronaviral particles, as published in the literature; Figure S1: NMR HSQC Spectra of Monomeric Griffithsin at pH 2.5 and pH 6.7; Figure S2: SDS-PAGE Gel demonstrating that all variants of Griffithsin ran at the size that was predicted based on their molecular weight; Figure S3: Control demonstrating Raji cells are not permissive to SARS-CoV-2 pseudoviral infection; Figure S4: Control demonstrating that Raji DC-SIGN+ cells facilitate trans-infection more efficiently than Raji Wild-Type cells, thereby demonstrating that DC-SIGN does appear to capture SARS-CoV-2 pseudovirions; Figure S5: Control demonstrating that mannan inhibits Raji DC-SIGN+ cell-mediated trans-infection in a dose-dependent manner; Figure S6: Representative diagrams of virions when cells are transfected with additional structural protein expression vectors; Figure S7: Raw direct and trans-infectivity for virus strains with additional structural proteins as well as virus titers of these additional structural protein strains; Figure S8: Depiction of spike capture ELISA and spike ELISA to quantify the levels of spike in Wild-Type, M, N, E, and MNE SARS-CoV-2 pseudotyped lentivirus samples; Figure S9: Depiction of how additional structural proteins did not affect pseudoviral strains’ propensity to undergo 3t3 DC-SIGN+ cell-mediated trans-infectivity as normalized to direct infection; Figure S10: Depiction of how additional structural proteins did not affect pseudoviral strains’ susceptibility to mannan inhibition of DC-SIGN-mediated trans-infection; Figure S11: Verifying that luciferase assays were performed within viable parameters like MOI and exposure time by demonstrating that neutralizing antibody against SARS-CoV-2 spike displayed consistent IC_50_ Values.
