# Supplementary material for: The Antiviral Activity of the Lectin Griffithsin against SARS-CoV-2 Is Enhanced by the Presence of Structural Proteins
Source: Viruses. 2023 Dec 18;15(12):2452. doi: 10.3390/v15122452 (PMC10747160; doi:10.3390/v15122452)
Supplement: Supplementary file 1 [file viruses-15-02452-s001.zip › viruses-2689479-supplementary.pdf]

Supplemental Table

Table S1. A summary of Wild-Type Grifithsin's antiviral capabilities against SARS-CoV-2 pseudotyped lentivirus versus coronaviral particles, as published in the literature.

Article	Reference Number	Source of Griffithsin	SARS-CoV-2 Viral Strain	Inhibitory Capabilities Against SARS-CoV-2 Pseudotyped Lentivirus or other in vitro model assessing Spike alone	Inhibitory Capabilities Against SARS-CoV-2 Coronaviral Particles
Alsaidi S, et. al. Griffithsin and Carrageenan Combination Results in Antiviral Synergy against SARS-CoV-1 and 2 in a Pseudoviral Model. Mar Drugs. 2021 Jul 26;19(8):418. doi: 10.3390/md19080418.	35	Expressed in *Nicotiana benthamiana* plants transduced with a tobacco mosaic virus vector expressing WT-GRFT.	Wild-Type:	Inhibition of SARS-CoV-2 pseudotyped lentiviral infection *in vitro* on HeLa cells expressing hACE2: EC50 = 20.6 µg/mL (1610 nM)	Not Reported
			D641G:	Inhibition of pseudoviral infection *in vitro* on HeLa cells expressing hACE2: EC50 = 37.6 µg/mL (2940 nM)	
			K417N /E484K/ N501Y*:	Inhibition of SARS-CoV-2 pseudotyped lentiviral infection *in vitro* on HeLa cells expressing hACE2: EC50 = 31.6 µg/mL (2750 nM)	
Cai Y, et. al. Griffithsin with A Broad-Spectrum Antiviral Activity by Binding Glycans in Viral Glycoprotein Exhibits Strong Synergistic Effect in Combination with A Pan-Coronavirus Fusion Inhibitor Targeting SARS-CoV-2 Spike S2 Subunit. Virol Sin. 2020 Dec;35(6):857-860. doi: 10.1007/s12250-020-00305-3.	37	Expressed in *Escherichia coli* BL21 (DE3) via pET expression vector.	Wild-Type:	Inhibition of SARS-CoV-2 pseudotyped lentiviral infection *in vitro* on HuH-7 cells: IC_{50} = 292.5 nM to 447.3 nM	Inhibition of coronaviral particle infection *in vitro* on VeroE6 cells: IC_{50} = 63 nM
			Wild-Type:	Inhibition of SARS-CoV-2 Spike mediated HuH-7 cell-cell fusion: IC_{50} = 322.9 nM	

Cai Y, et. al. A bivalent protein targeting glycans and HR1 domain in spike protein potently inhibited infection of SARS-CoV-2 and other human coronaviruses. Cell Biosci. 2021 Jul 8;11(1):128. doi: 10.1186/s13578-021-00638-w.	38	Expressed in *Escherichia coli* BL21 (DE3) via pET expression vector	Wild-Type:	Inhibition of SARS-CoV-2 pseudotyped lentiviral infection *in vitro* on HuH-7 cells: IC_{50} = 510.8 nM	Inhibition of coronaviral particle infection on *in vivo* mice models: IC_{50} = 100.1 nM
			Wild-Type:	Inhibition of SARS-CoV-2 pseudotyped lentiviral infection *in vitro* on Caco-2 cells: IC_{50} = 885.7 nM	
			Wild-Type:	Inhibition of SARS-CoV-2 Spike mediated cell-cell fusion: IC_{50} = 322.9 nM	
Ahan RE, et. al. A Highly Potent SARS-CoV-2 Blocking Lectin Protein. ACS Infect Dis. 2022 Jul 8;8(7):1253-1264. doi: 10.1021/acsinfecdis.2c00006.	39	Expressed in *Escherichia coli* BL21 (DE3) via pET expression vector	Wild-Type:	Binding affinity of Spike to Grft protein determined by Isothermal Titration Calorimetry (ITC): Kd = 9900 nM	
			Wild-Type:	Binding affinity of Spike to Grft protein determined by Quartz Crystal Microbalance with Dissipation monitoring (QCM-D): Kd = 1600 nM	Inhibition of coronaviral particle infection *in vitro* on VeroE6 cells: IC_{50} = 33.2 nM

			Delta:		Inhibition of coronaviral particle infection *in vitro* on VeroE6 cells: IC_{50} = 34.0 nM
			Omicron:		Inhibition of coronaviral particle infection *in vitro* on VeroE6 cells: IC_{50} = 5.4 nM
Borhani SG, et al. An approach to rapid distributed manufacturing of broad spectrum anti-viral griffithsin using cell-free systems to mitigate pandemics. N Biotechnol. 2023 Sep 25;76:13-22. doi: 10.1016/j.nbt.2023.04.003.	40	Cell-free expression with plant and microbial proteins via pET expression vector	Not explicitly specified; assumed to be Wild-Type based off of cited literature within the publication	Inhibition of SARS-CoV-2 pseudotyped Murine Leukemia Virus particles *in vitro* on HEK-293T cells overexpressing human ACE2. Wild-Type Griffithsin: IC_{50} = 510 nM	Not Reported

				Inhibition of SARS-CoV-2 pseudotyped Murine Leukemia Virus particles *in vitro* on HEK-293T cells overexpressing human ACE2. M78Q Griffithsin: IC_{50} = 324 nM	

Supplemental Figures

A.)

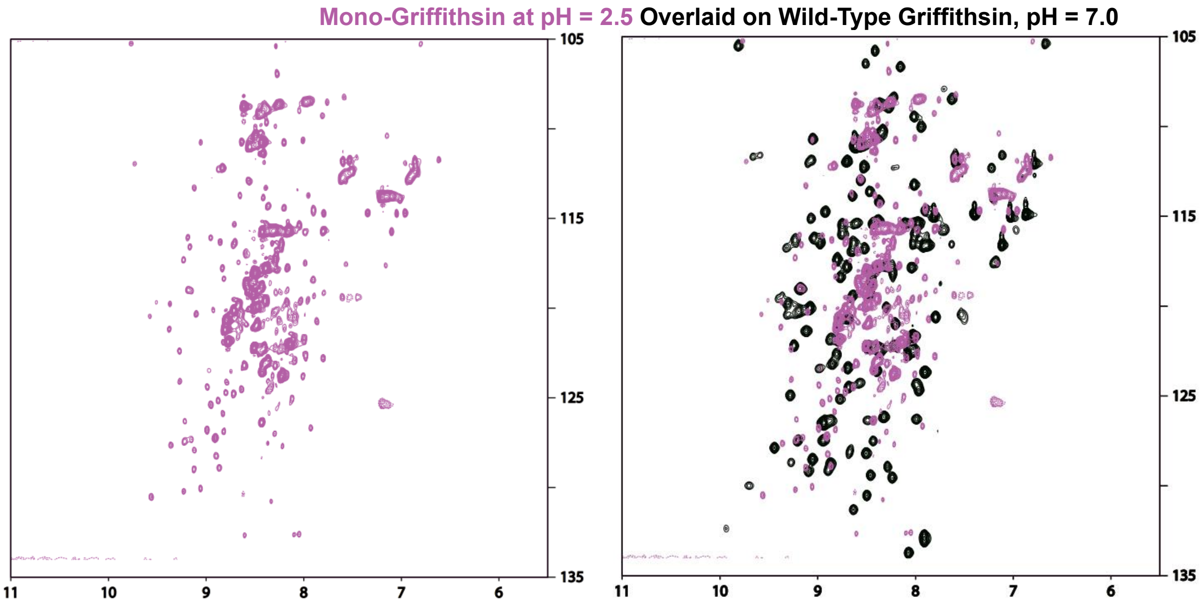

B.)

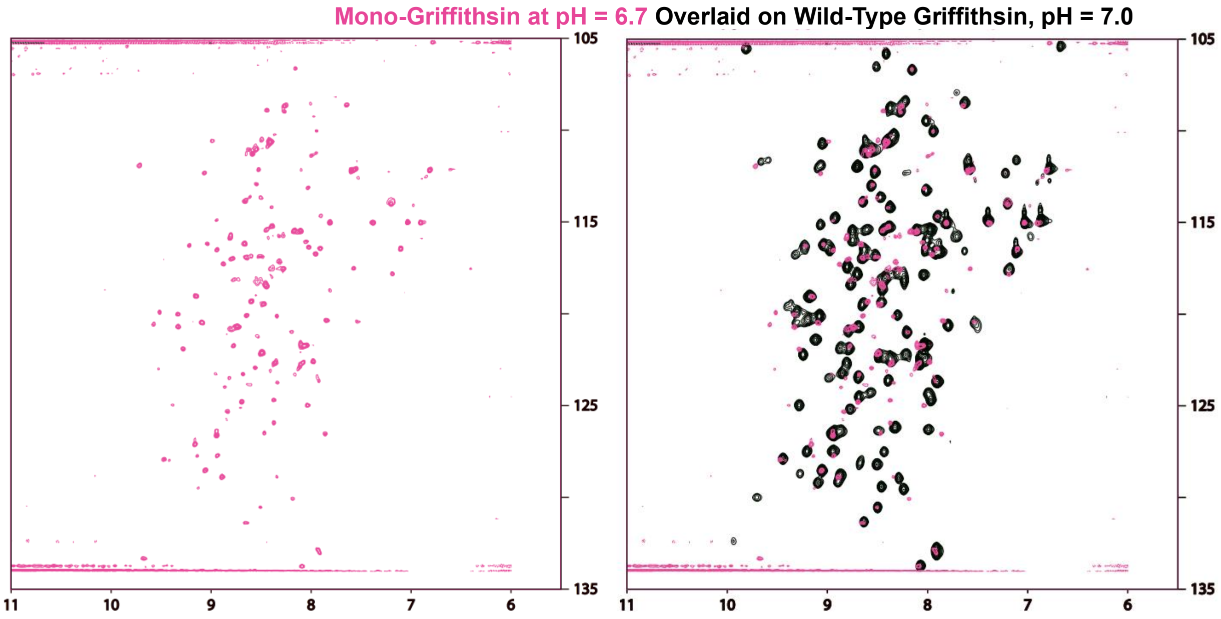

Figure S1: A.) The NMR HSQC spectrum of Mono-Griffithsin at pH 2.5 (purple). The variation in peak intensity indicates that the protein is not stable and is likely forming aggregates. When the spectrum is overlaid on WT-Grft taken at pH 7.0, several peaks look shifted (as would be expected due to changes in pH between the two spectra). **B.)** The NMR HSQC spectrum of Mono-Griffithsin at pH 6.7 (pink). The protein precipitated during this run, as hinted at by the greatly reduced peak intensity on the spectrum. However, the majority of the peaks overlay

with Wild-Type Griffithsin, indicating that the subset of Mono-Griffithsin that remains in solution is likely well-folded and functional. However, Mono-Griffithsin appeared to have a saturation limit beneath 1 μM in this solution, making it unsuitable for further assessment.

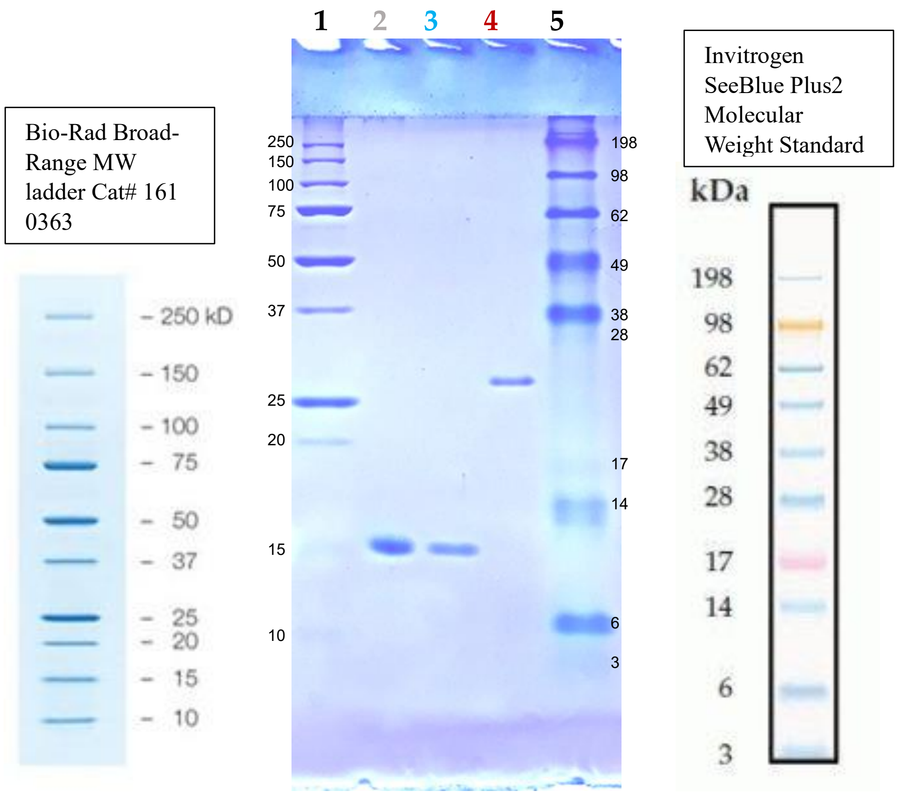

Lane 1: Bio-Rad Broad-Range MW ladder Cat# 161 0363
Lane 2: Wild-Type Griffithsin (WT-Grft)
Lane 3: M78Q-Griffithsin (Q-Grft)
Lane 4: Griffithsin-Linker-Griffithsin "Onearmed" Subdomain A Wild-Type, Subdomain B 3A (Grft-L-Grft 3A)
Lane 5: Invitrogen SeeBlue Plus2 Molecular Weight Standard

Expected size of lectin proteins:
Wild-Type Griffithsin: 14.69 kDa
M78Q-Griffithsin: 14.68 kDa
Griffithsin-Linker-Griffithsin "Onearmed" Subdomain A Wild-Type, Subdomain B 3A: 28.37 kDa

Figure S2: Representative SDS-PAGE Gel demonstrating that all three Griffithsin variants appeared to be the correct size after purification.

A.)

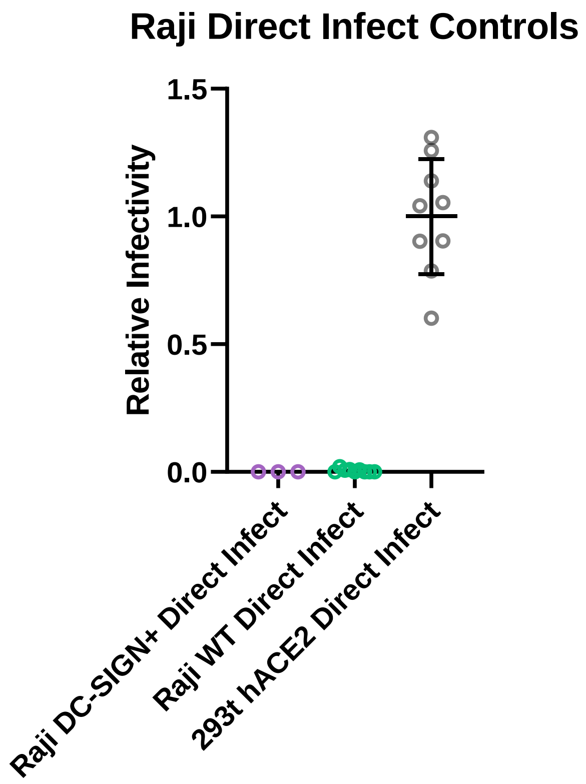

B.)

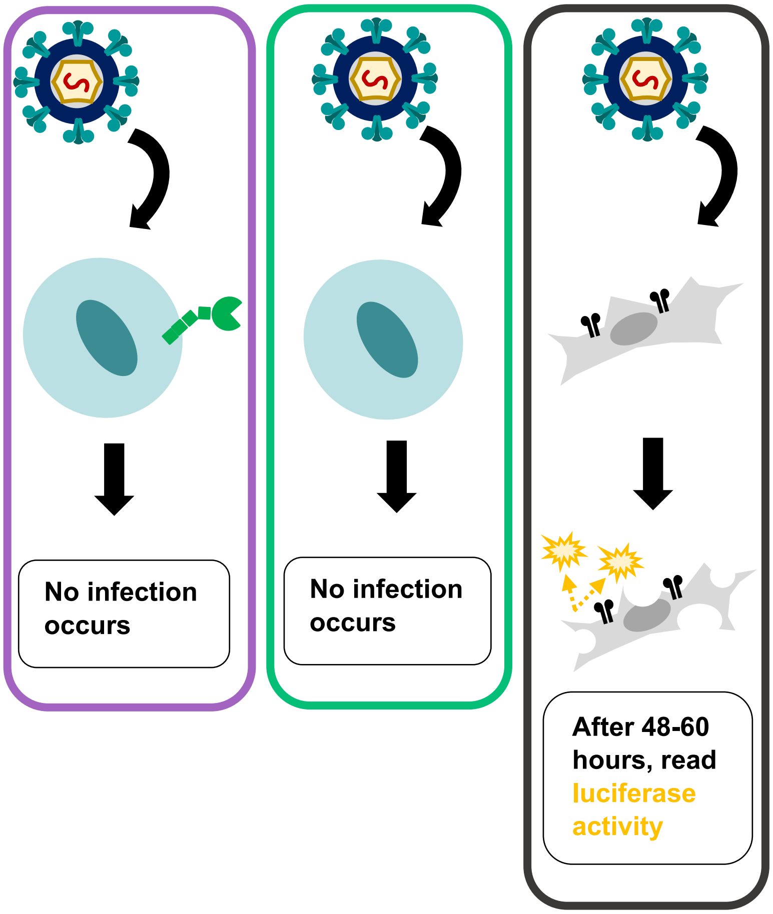

Figure S3: Verification that Raji WT and Raji DC-SIGN+ cells were not permissible to SARS-CoV-2 pseudoviral infection. **A.)** The comparing Direct infection signal of Raji DC-SIGN+ Cells and Raji WT Cells exposed to SARS-CoV-2 pseudotyped virions to the Direct infection signal of 293T hACE2+ cells shows that they did not allow for infection. **B.)** Schematic depictions of Direct infection assay.

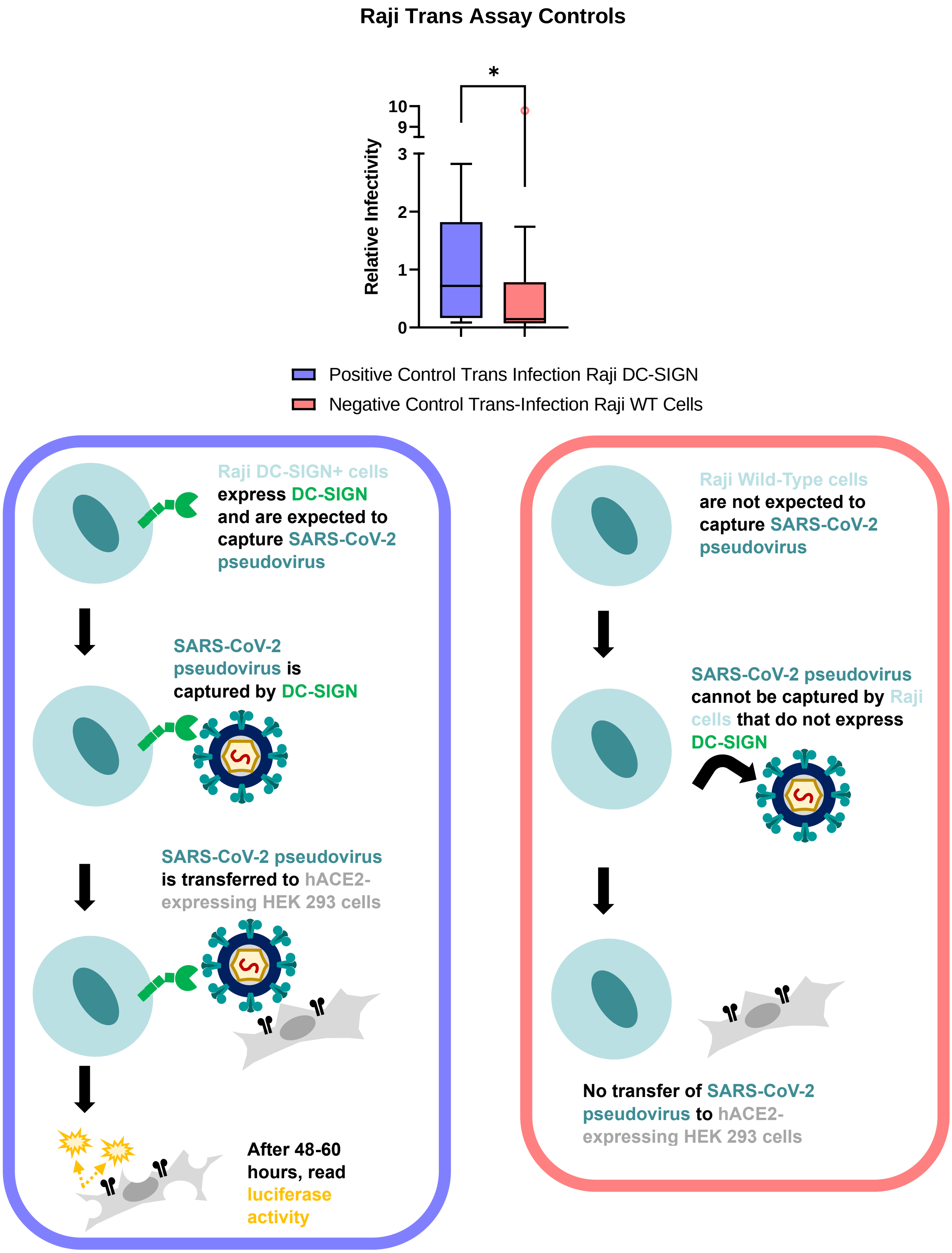

Raji Trans Assay Controls
*
Relative Infectivity
10
9
3
2
1
0
Positive Control Trans Infection Raji DC-SIGN
Negative Control Trans-Infection Raji WT Cells
Raji DC-SIGN+ cells express DC-SIGN and are expected to capture SARS-CoV-2 pseudovirus
SARS-CoV-2 pseudovirus is captured by DC-SIGN
SARS-CoV-2 pseudovirus is transferred to hACE2-expressing HEK 293 cells
After 48-60 hours, read luciferase activity
Raji Wild-Type cells are not expected to capture SARS-CoV-2 pseudovirus
SARS-CoV-2 pseudovirus cannot be captured by Raji cells that do not express DC-SIGN
No transfer of SARS-CoV-2 pseudovirus to hACE2-expressing HEK 293 cells

Figure S4: Raji assay controls were summarized to see the difference between viral capture for Raji WT cells versus Raji DC-SIGN+ cells. Plot depicts medians with values between the 10th to 90th percentile. Despite the wide range of Relative Infectivity values for both Positive Control and Negative Control samples, comparing the median and 10th-90th percentile values for both groups shows that Raji DC-SIGN+ cells appear to facilitate more Trans-infectivity than Raji WT cells. The high variability in data values was endemic to nearly all of our assays we performed with Raji cells, which was another key driving factor that led us to want to verify our results with 3t3 cells. * indicates a p-value < 0.1 on a Mann-Whitney T-test.

A.)

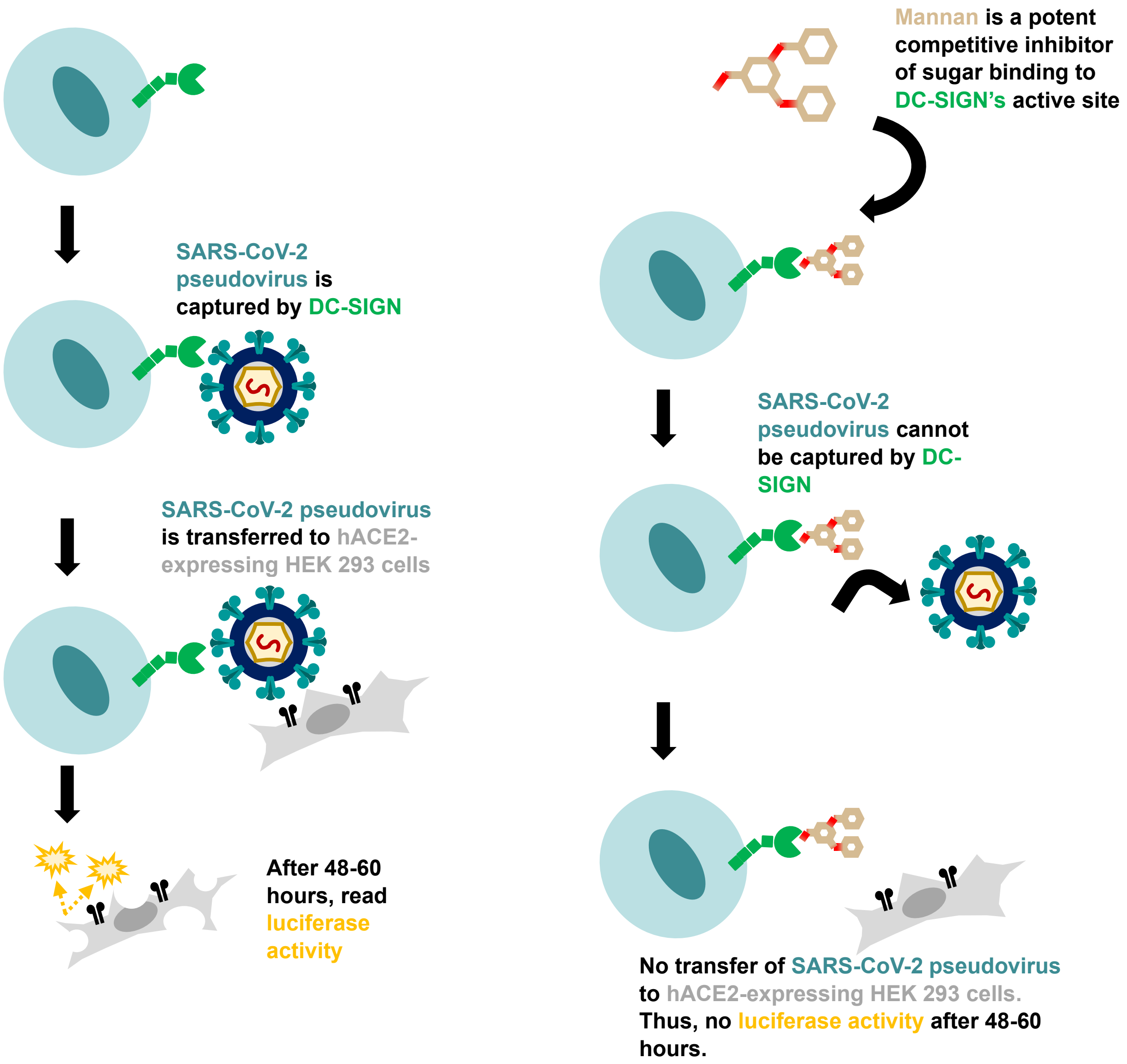
Mannan is a potent competitive inhibitor of sugar binding to DC-SIGN's active site
SARS-CoV-2 pseudovirus is captured by DC-SIGN
SARS-CoV-2 pseudovirus cannot be captured by DC-SIGN
SARS-CoV-2 pseudovirus is transferred to hACE2-expressing HEK 293 cells
After 48-60 hours, read luciferase activity
No transfer of SARS-CoV-2 pseudovirus to hACE2-expressing HEK 293 cells. Thus, no luciferase activity after 48-60 hours.

B.)

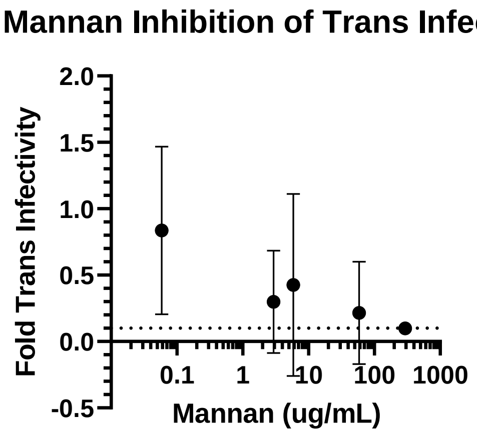
Mannan Inhibition of Trans Infection
Fold Trans Infectivity
2.0
1.5
1.0
0.5
0.0
-0.5
0.1
1
10
100
1000
Mannan (ug/mL)

Figure S5: Verification that mannan acts as an inhibitor of Raji DC-SIGN+ Cell-mediated Trans-infection. A.) Schematic diagram of how mannan inhibits Raji DC-SIGN+ mediated Trans-infection. **B.)** Results of Mannan inhibition of a Raji DC-SIGN+ mediated Trans-infection assay. This was performed to verify that DC-SIGN was indeed the cell surface protein that was responsible for capturing virions during Trans-infection.

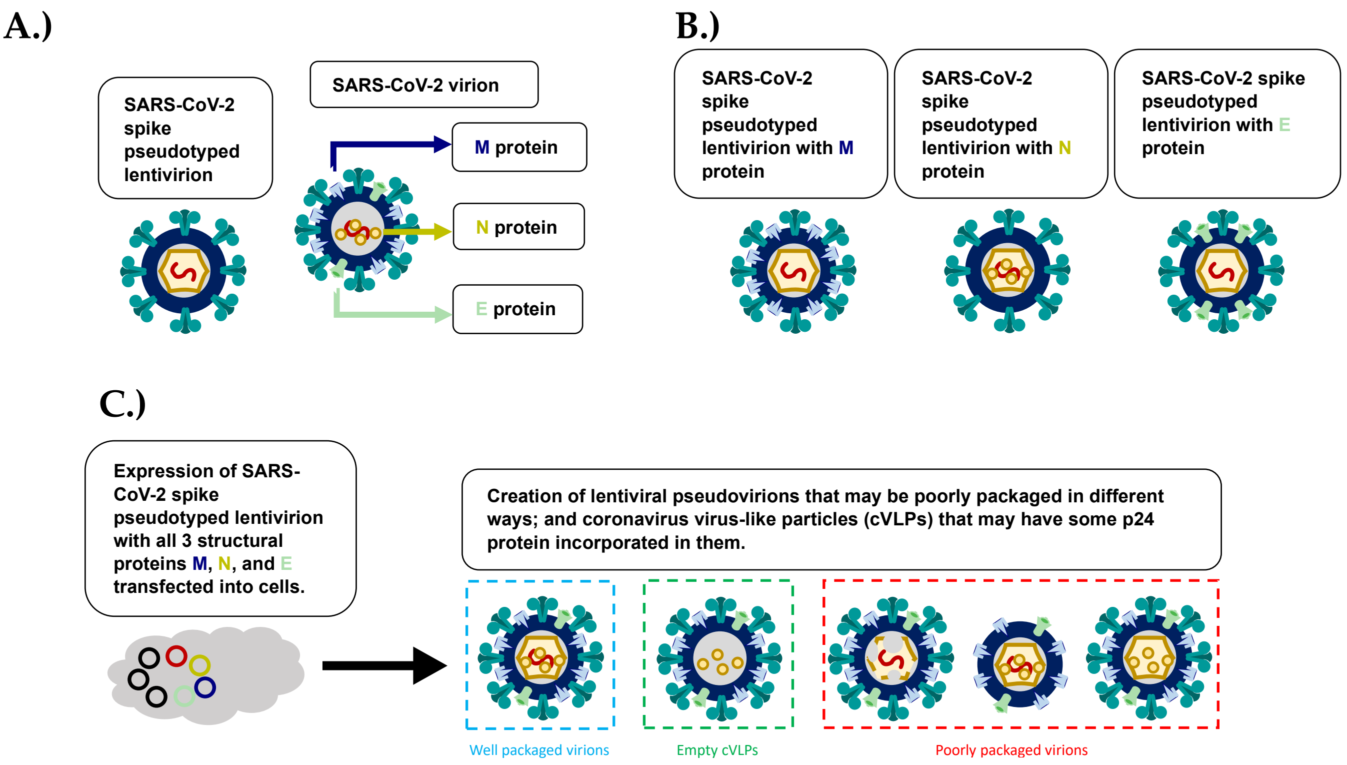

Figure S6. Representative diagrams of virions described in this chapter. A.) Comparison of the exterior of SARS-CoV-2 Spike pseudotyped lentivirions to SARS-CoV-2 coronaviral particles. **B.)** Also depicted are representations of the three coronavirus strains where each of the 3 structural proteins were added in turn. The N protein stays on the interior of the virion while M and E are expressed on the surface. **C.)** Simplified depiction of the transfection process to create pseudovirions; structural protein vectors are depicted in blue, yellow and mint green while the transfer vector is shown in red. Other lentiviral packaging vectors are depicted in black. Transfected cells can produce well-packaged MNE lentiviral pseudovirions (in teal blue box). In addition, expression of M, N, and E proteins can form coronaviral Virus-Like Particles (cVLPs, in green box). These cVLPs and other noninfectious poorly-packaged viral particles (in red box) may incorporate some p24, which can increase the apparent viral titer on p24 ELISA assays.

A.)

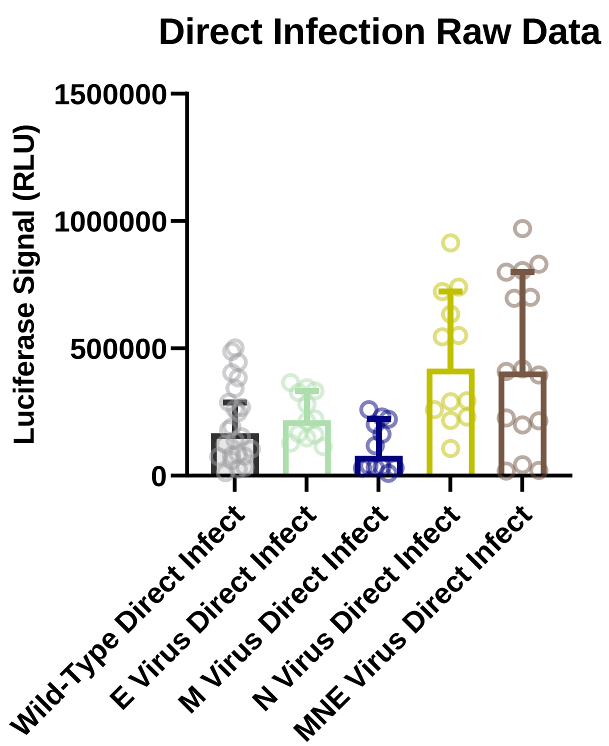

B.)

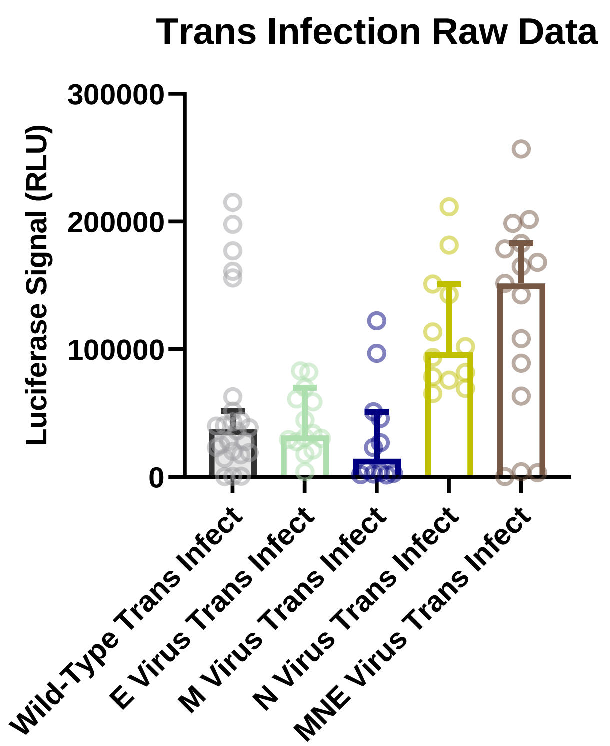

C.)

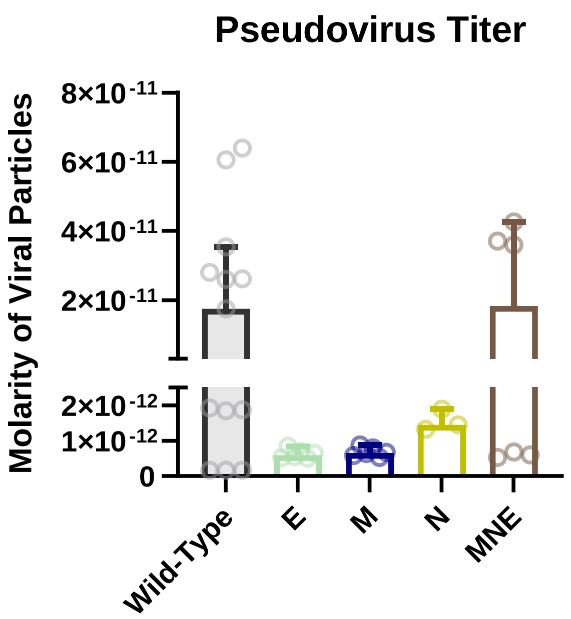

Figure S7. A.) Depiction of the raw hACE2-mediated Direct Infection Luciferase signal for all SARS-CoV-2 pseudoviral strains. **B.)** Depiction of the raw 3t3 DC-SIGN+ cell-mediated Direct

Infection Luciferase signal for all SARS-CoV-2 pseudoviral strains. **C.)** Pseudoviral titer as measured by p24 ELISA assay. At least two different batches of virus were made for each variant. Plots depict medians with 95% Confidence Interval.

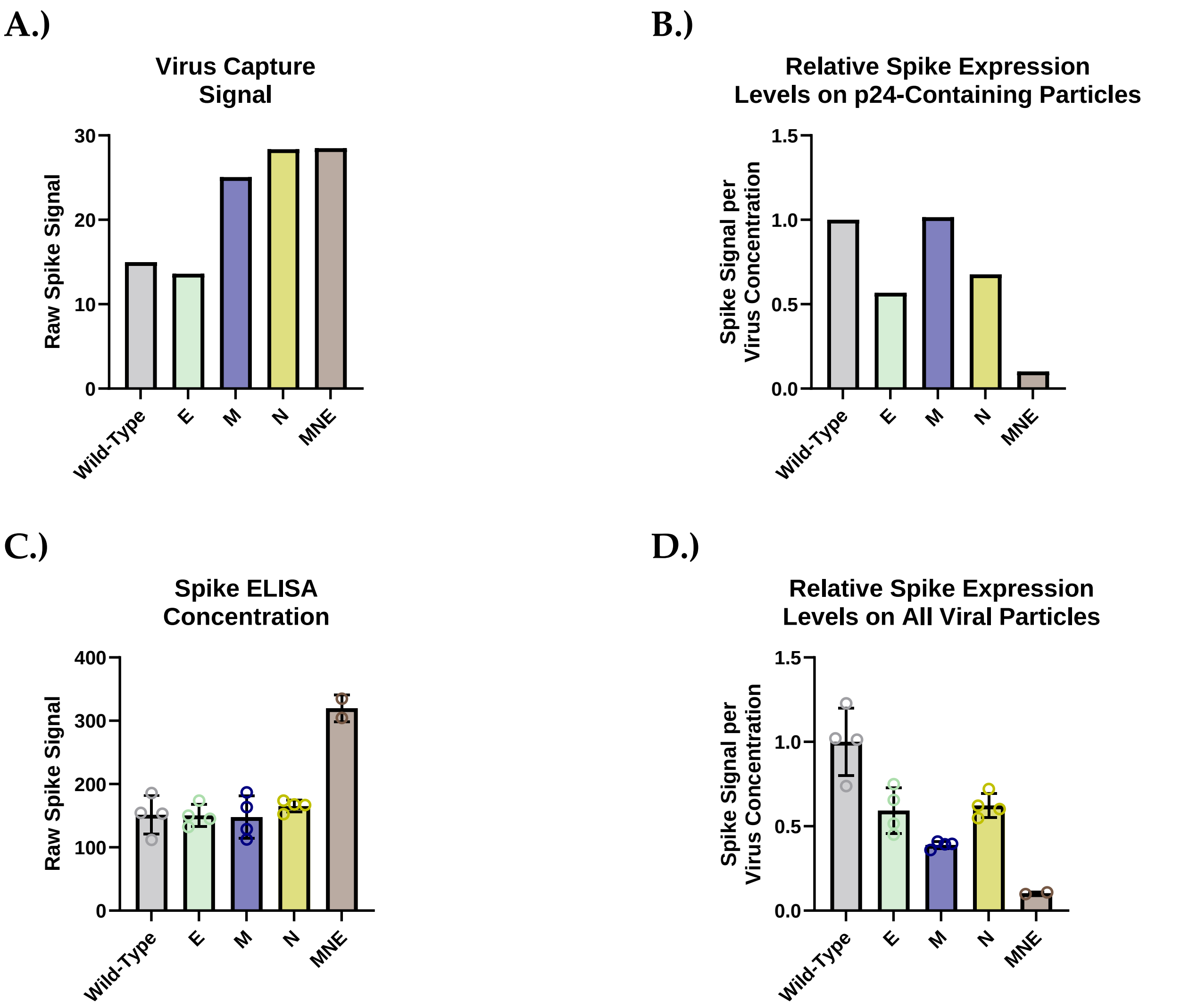

Figure S8. A.) Depiction of the raw Spike protein concentration for all SARS-CoV-2 pseudoviral strains on a Spike Capture ELISA assay. **B.)** Depiction of the relative Spike protein levels per virion for all SARS-CoV-2 pseudoviral strains. **C.)** Depiction of the raw Spike protein concentration in each pseudoviral strain calculated via Spike Direct ELISA assay. **D.)** Depiction of the relative Spike protein levels per virus for all SARS-CoV-2 pseudoviral strains as calculated via Spike Direct ELISA assay. The data in **A.)** reflects the raw number of pseudoviral particles that contain a p24 capsid and also have Spike protein on their surface. In contrast, the data in **C.)** solely reflects the raw number of pseudoviral particles that have Spike protein on their surface. As we can see, the M and N strains appeared to have about a 70% higher proportion of viral particles that contain p24 in their interior when compared to the Wild-Type pseudoviral strain. The MNE sample appeared to contain a higher number of Spike-expressing and p24-incorporating particles overall. The data in **B.)** reflects the relative proportion of viral particles that express both p24- and Spike- for each pseudoviral sample. The data in **D.)** reflects the relative proportion of Spike-containing particles that are in the pseudoviral sample.
The data from **B.)** and **D.)** indicate that the presence of M protein decreases the average Spike expression, but for well-packaged viral particles that contain p24, the level of Spike protein

expression remains very similar to the Wild-Type Strain. The presence of N and E proteins appeared to cause the average Spike expression to decrease by about 30%-45% per particle, while the presence of all 3 structural proteins (MNE) decreased the average Spike protein expression per particle by about 90%. However, the high infectivity of these samples (Figure S7) indicated to us that the decrease in the average level of Spike protein expression was likely not because of significantly decreased Spike expression on well-packaged viral particles, but rather because the additional structural proteins increased the number of poorly-packaged coronavirus-like particles in each sample.

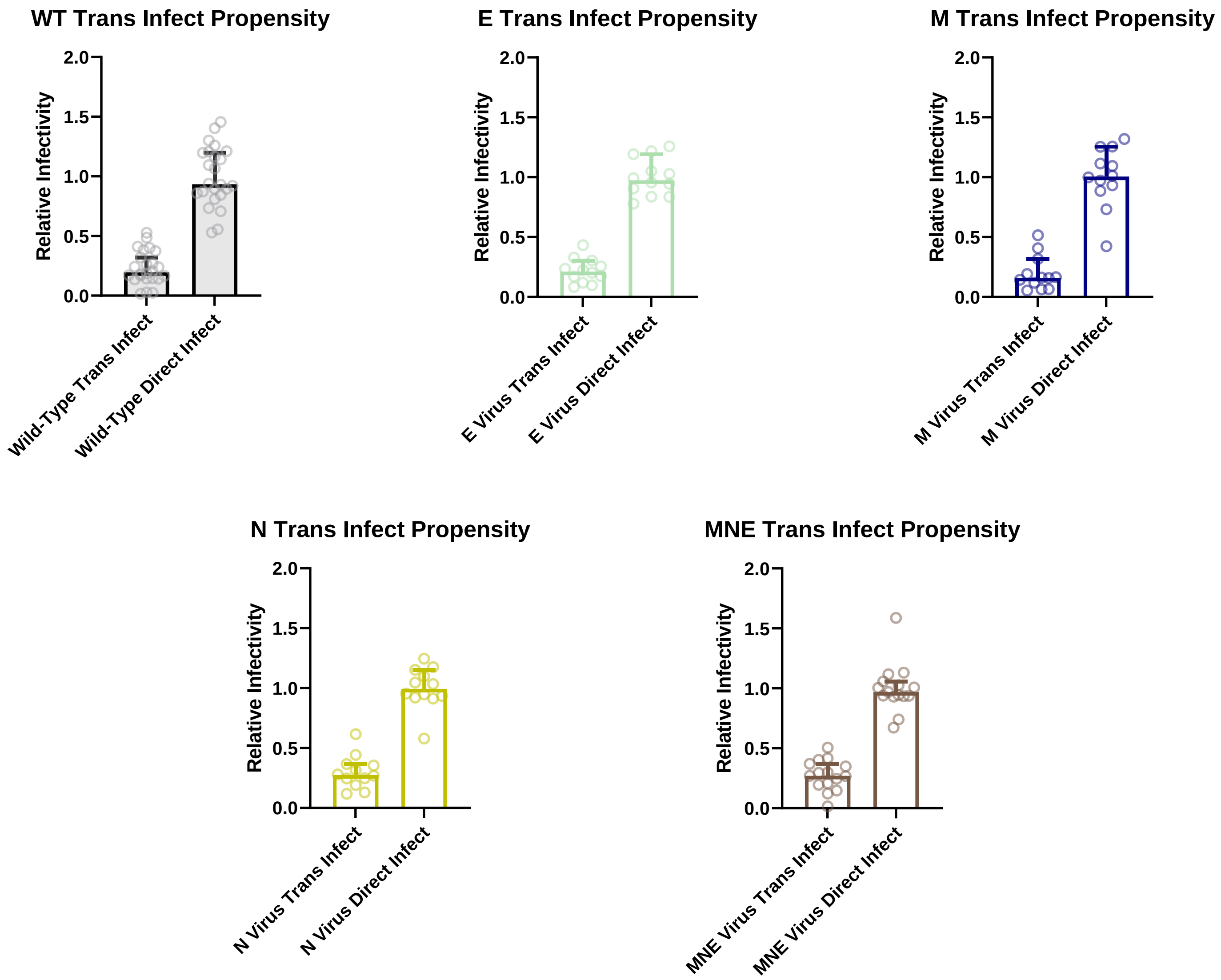

Figure S9: Comparisons depicting additional structural protein pseudoviral strains' propensity to undergo 3t3 DC-SIGN+ cell mediated Trans Infectivity as normalized to Direct Infection.

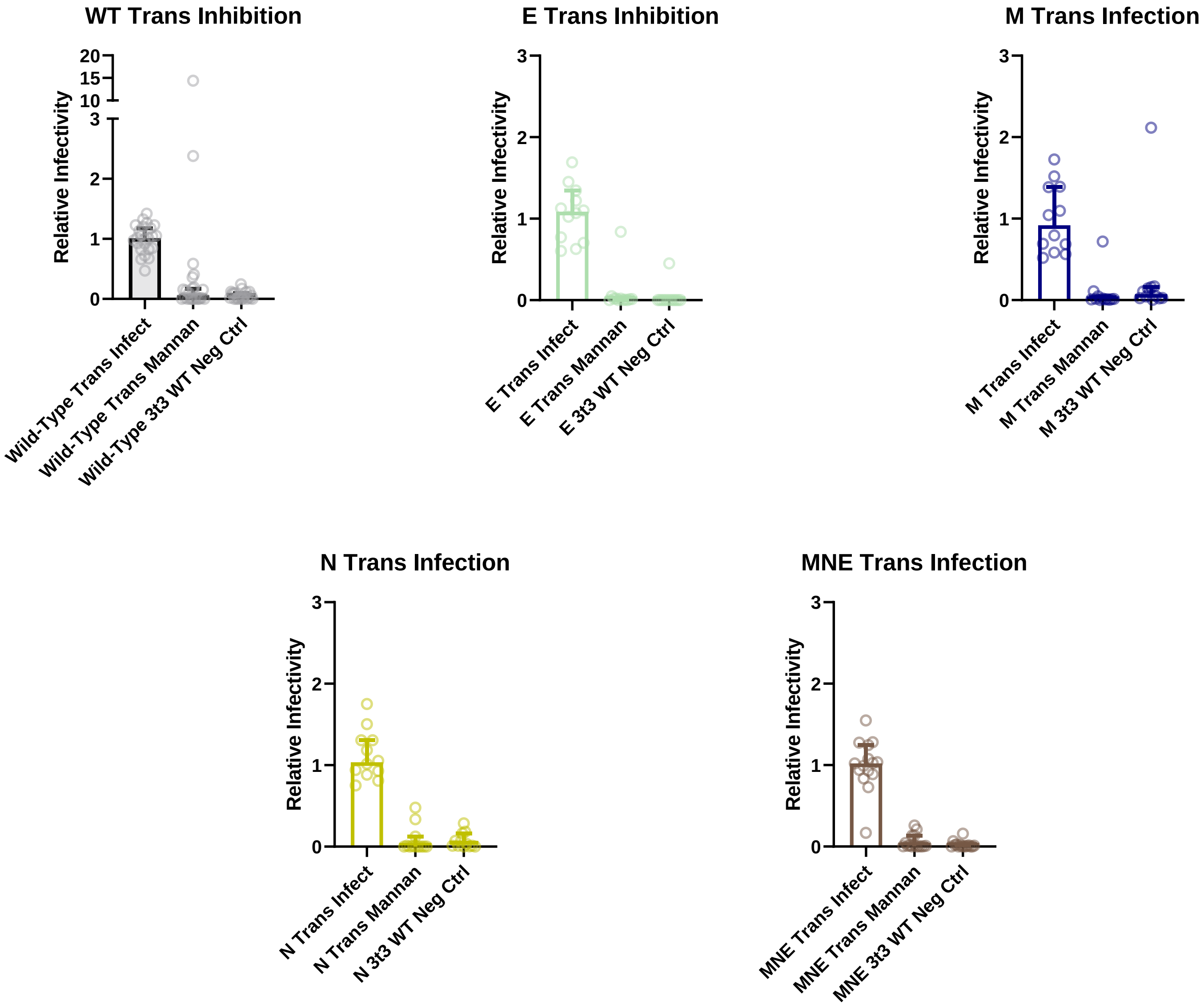

Figure S10: Comparisons depicting additional structural protein pseudoviral strains' susceptibility to mannan on 3t3 cell assay. For each sample, virus infectivity per molar was normalized to each strain's Trans-infection signal.

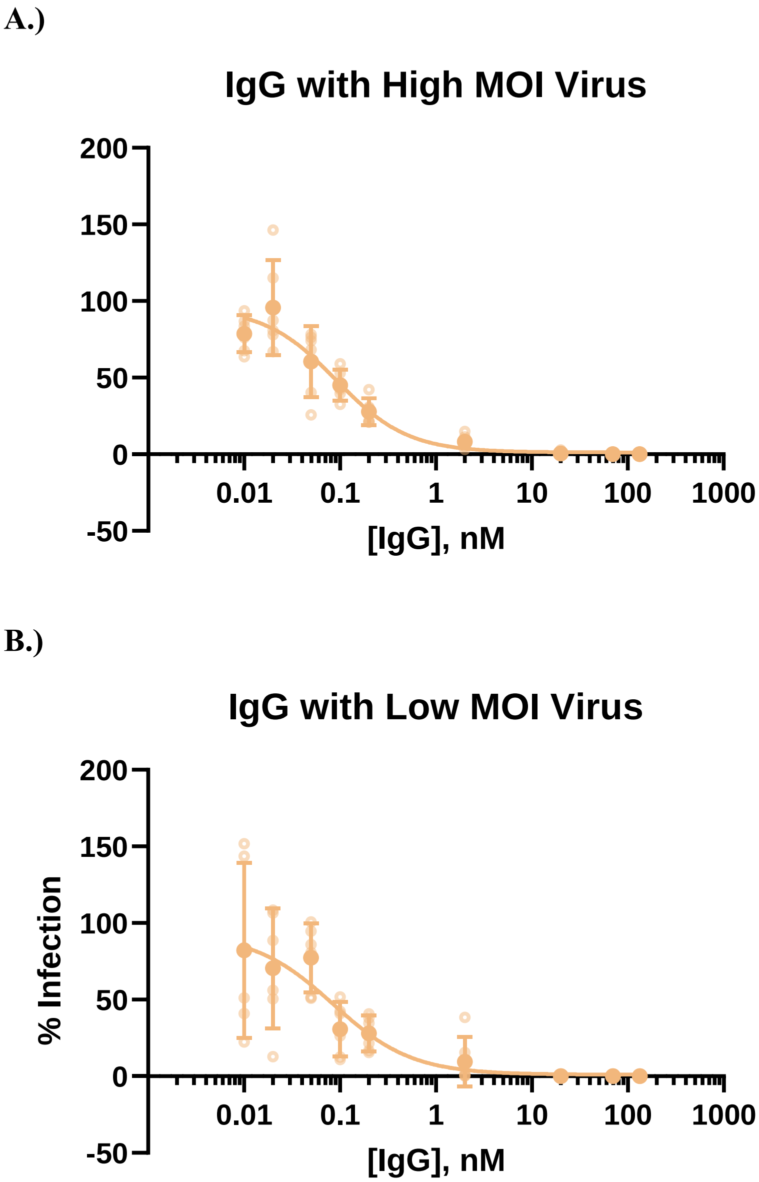

Figure S11: Verifying that luciferase assays were performed within viable parameters like MOI and exposure time by demonstrating that neutralizing antibody against SARS-CoV-2 Spike displayed consistent IC50 Values. Comparison of inhibition curves when we used the SARS-CoV-2 anti Spike protein Neutralizing IgG (SinoBiological Cat # 40592-R001) on our infectivity assay at undiluted virus (High MOI) and tenfold diluted virus (Low MOI). **A.)** Infectivity curves when using undiluted pseudoviral samples at a High MOI. IC_{50} = 0.085 nM **B.)** Infectivity curves when using tenfold diluted pseudoviral samples. IC_{50} = 0.074 nM.
